# Assessing Structural, Thermal, and Mechanical Properties of Zirconium‐Based Metal‐Organic Frameworks via DFTB3/3OB MD Simulations: A Benchmark Beyond UiO Frameworks

**DOI:** 10.1002/jcc.70536

**Published:** 2026-10-28

**Authors:** Armin Penz, Thomas S. Hofer

**Affiliations:** ^1^ Department of General, Inorganic and Theoretical Chemistry University of Innsbruck Innsbruck Austria

## Abstract

The exceptional stability and broad application potential of zirconium‐based metal‐organic frameworks call for reliable atomistic methods capable of describing their structural, thermal, and mechanical properties. In this work, we benchmark the recently introduced extension of the DFTB3/3OB parameter set for the transition metal zirconium by evaluating a representative set of Zr‐containing metal‐organic frameworks spanning different secondary building units and topologies, namely UiO‐66, UiO‐67, MOF‐801, MIL‐140A, and MIL‐163. Equilibrium lattice parameters, X‐ray diffraction patterns, thermal expansion coefficients, and volumetric stiffness measures have been computed from DFTB3/3OB molecular dynamics simulations and compared to literature reference data. The results have been further assessed using hybrid DFT (HSEsol) reference calculations and contextualized through an independent comparison with neural‐network‐based MACE‐DAC‐1 simulations. Across the benchmark set, DFTB3/3OB provides a consistent structural description and reproduces the dominant experimental diffraction features, while the deviations of the lattice parameters remain small. DFTB3/3OB correctly reproduces the negative thermal expansion behavior of the UiO frameworks and predicts positive thermal expansion for MOF‐801; for the investigated MIL‐type systems, the observed anisotropic expansion highlights a stronger method dependence. For the cubic frameworks UiO‐66 and UiO‐67, the bulk moduli obtained via DFTB3/3OB fall within reported reference ranges and generally indicate intermediate stiffness relative to MACE‐DAC‐1 and HSEsol. For the non‐cubic MIL‐140A and MIL‐163, the corresponding MD‐derived fixed‐shape isotropic volumetric moduli provide a consistent basis for comparison between DFTB3/3OB and MACE‐DAC‐1. Overall, the presented results support the applicability and transferability of DFTB3/3OB to molecular dynamics simulations of zirconium‐based MOFs beyond the prototypical UiO frameworks.

## Graphical abstract

DFTB3/3OB is assessed across structurally diverse Zr‐based MOFs through molecular dynamics simulations. Comparison of the resulting lattice parameters, X‐ray diffraction patterns, thermal expansion coefficients, and volumetric stiffness measures with literature data and HSEsol reference calculations highlights its applicability beyond the prototypical UiO frameworks.

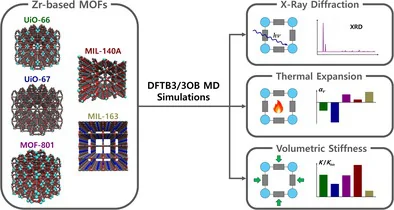

## 1 Introduction

Recent advances in materials science have highlighted metal‐organic frameworks (MOFs) as a transformative class of porous materials, distinguished by their exceptional versatility and structural tunability [1, 2, 3, 4]. The field's profound impact was underscored by the 2025 Nobel Prize in Chemistry, awarded jointly to Susumu Kitagawa [5, 6], Richard Robson [7, 8], and Yaghi [9, 10] for their pioneering development of these framework compounds. MOFs are coordination networks constructed from metal clusters or ions bridged by organic linkers. As such, they possess a modular architecture that allows for precise control over pore size, topology, and chemical functionality, a design principle central to reticular chemistry [11]. This unique “molecular building block” approach endows MOFs with extraordinarily high surface areas and porosity, often exceeding those of traditional storage materials such as zeolites, activated carbons, and mesoporous silica [12, 13]. Consequently, they have been recognized as critical candidates for a wide array of applications, most notably in energy storage [14, 15, 16], gas separation [17, 18] and heterogeneous catalysis [19, 20, 21], while showing increasing promise in biomedical fields like targeted drug delivery [22, 23] and biochemical sensing [24, 25]. However, despite this broad potential, many MOFs exhibit limited stability under elevated temperatures, in aqueous environments or under mechanical stress compared to zeolites [26]. This limitation has motivated the exploration of alternative inorganic nodes and framework designs that offer enhanced structural stability.

In this context, zirconium‐based metal‐organic frameworks have emerged as a particularly robust subclass. The archetypal *Universitetet i Oslo* (UiO) family, introduced by Lillerud and co‐workers in 2008 [27], provides a landmark example of frameworks that combine high porosity with exceptional stability [28, 29]. This increased tolerance to external stimuli can be attributed to the composition of the secondary building unit (SBU), which consists of a $Zr_{6}O_{4}{\left(\text{OH}\right)}_{4}^{12+}$ cluster that can be coordinated by up to 12 organic linkers. In this structure, the zirconium cations form an octahedron, the faces of which are capped by oxide and hydroxide anions. Many other zirconium‐containing MOFs feature the same inorganic node, including, for example, NU‐1101 to NU‐1104 [30], MOF‐801, MOF‐802, MOF‐805, MOF‐806, MOF‐808, MOF‐812 [31], and PCN‐223 to PCN‐225 [32, 33, 34]. In contrast, some MOFs are based upon distinct zirconium clusters, several of which exhibit even higher hydrothermal and mechanical stability than the UiO‐type MOFs, such as MIL‐140A to MIL‐140D [35]. Beyond its structural advantages, zirconium is also present in the human body at trace levels (approximately 420 mg in total) [36] and can be readily eliminated [37]. These characteristics support the biocompatibility of zirconium and underpin the increasing focus on Zr‐based metal‐organic frameworks for biomedical applications. Such applications include their use as carriers for anticancer drugs [38, 39], platforms for the detection of cancer biomarkers [40, 41, 42], and agents for the detoxification of arsenic [43] and opioid poisoning [44].

As interest in metal‐organic frameworks continues to grow, computational methods have become an essential tool for elucidating their structure–property relationships. One prominent strategy is to employ high‐level approaches, such as post‐Hartree‐Fock methods and density functional theory [45, 46, 47, 48, 49]. Although these methods offer high accuracy in most cases, their computational cost becomes prohibitive for large systems and when applied in the context of molecular dynamics (MD) simulations. Classical force field approaches, on the other hand, enable fast, accurate, and scalable descriptions of pristine MOF systems [50, 51, 52, 53, 54]. However, commonly used conventional force fields exhibit limited performance in accurately describing guest‐host interactions and adsorbate‐induced deformations in metal‐organic frameworks [55, 56, 57]. In this context, the semi‐empirical DFTB3 method [58] represents an attractive intermediate approach, substantially reducing the computational demand relative to conventional DFT while retaining an explicit, self‐consistent quantum‐chemical description of the electronic structure. Utilizing the well‐established 3OB set of parameters has been shown to yield accurate results and valuable insights for numerous MOFs, as well as for complex guest@MOF systems [59, 60, 61, 62, 63, 64]. More recently, machine‐learning interatomic potentials have enabled atomistic simulations at substantially reduced computational cost while approaching DFT‐level accuracy within the domains represented by their training data [65, 66, 67]. DFTB3 nevertheless remains complementary to such approaches by providing direct access to electronic structure information and self‐consistent charge redistribution without requiring retraining for each new chemical environment, provided that the relevant elemental interactions are covered and validated by the parameter set. With the recent extension of the 3OB parameter set to include the transition metal zirconium [68], the accessible chemical space has been expanded to Zr‐containing systems, including Zr‐based MOFs and corresponding host‐guest applications.

The aim of this work is to thoroughly benchmark the performance and accuracy of this extension with respect to Zr‐based MOFs. For this purpose, the thermal expansion coefficients as well as bulk moduli for the cubic frameworks and fixed‐shape isotropic volumetric moduli for the non‐cubic frameworks have been calculated for the five Zr‐containing metal‐organic frameworks UiO‐66, UiO‐67, MOF‐801, MIL‐140A, and MIL‐163 and compared with literature data. In addition, lattice parameters and X‐ray diffraction (XRD) patterns have been computed for MOF‐801, MIL‐140A, and MIL‐163 and likewise evaluated against reported values. For UiO‐66 and UiO‐67, the comparison of lattice parameters and XRD patterns has already been presented in our previous work [68]. Furthermore, calculations have been performed using the machine learning force field (MLFF) MACE‐DAC‐1 [67], which has been developed for direct air capture (DAC) applications by fine‐tuning MACE‐MP‐0 [69] for CO_2_ and H_2_O interactions in chemically diverse MOFs. On the GoldDAC test set, comprising 312 gas@MOF configurations across 26 different MOFs, MACE‐DAC‐1 achieves mean absolute errors of 0.188 eV for interaction energies and 0.098 eV Å^−1^ for forces relative to DFT reference data [67]. While this validation establishes MACE‐DAC‐1 as a relevant MOF‐focused MLFF within its intended adsorption domain, it has not been specifically validated for the thermomechanical properties investigated in the present work. Its predictions are therefore included as an independent MLFF comparison rather than as reference data. Complementary high‐level calculations using the well‐established HSEsol hybrid functional [70] have been performed to provide an additional reference.

## 2 Methodology

This section outlines the computational setup, the MD simulation workflow, and the analysis strategies used to determine thermal expansion coefficients as well as bulk moduli for the cubic frameworks and fixed‐shape isotropic volumetric moduli for the non‐cubic frameworks. All DFTB calculations in this study have been carried out using the DFTB+ program package version 24.1 [71], employing the 3OB parameter set [68, 72, 73]. To ensure an accurate description of short‐range interactions involving hydrogen, an exponent ζ of 4.0 has been employed in the associated damping factor [74]. The convergence criterion for the self‐consistent charge (SCC) iterations has been set to 10^−5^, corresponding to an energy convergence tighter than 10^−8^ Hartree. For DFTB3/3OB and MACE‐DAC‐1, dispersion interactions have been accounted for using the DFT‐D3 correction scheme proposed by Grimme et al. [75] in combination with Becke–Johnson damping [76]. All calculations at MACE‐DAC‐1 level of theory have been executed via the atomic simulation environment (ASE) framework (v3.29.0) [77].

All molecular dynamics simulations have been performed via the PQ‐0.5.3 software [78]. For integration of the equations of motion, the velocity Verlet algorithm [79] has been utilized. The temperature has been controlled via the Bussi–Donadio–Parrinello thermostat [80] with a relaxation time of 0.1 ps, while pressure coupling has been achieved using the Berendsen manostat [81] with a relaxation time of 10 ps.

X‐ray diffraction (XRD) patterns have been computed using RIETAN‐FP [82] as implemented in the program VESTA‐3.5.7 [83]. For each system, 20,000 configurations have been extracted at uniform time intervals from a 200 ps unconstrained (i.e., without applying bond constraints) MD simulation trajectory and used for XRD calculations. The resulting diffraction patterns have been averaged to yield a representative XRD profile for each framework. To enhance comparability between XRD patterns, peak broadening has been applied using Gaussian kernel density estimation (KDE) with a standard deviation of 0.07°.

The initial structures of UiO‐66 [84], UiO‐67 [84], MOF‐801 [31], MIL‐140A [85], and MIL‐163 [86] have been obtained from their respective published sources. Subsequently, full geometry optimizations of both atomic positions and unit cell parameters have been performed using cell constraints chosen according to the symmetry of the respective frameworks. For the cubic UiO‐66, UiO‐67, and MOF‐801 systems, isotropic cell scaling has been employed, preserving a = b = c and α = β = γ = 90°. In contrast, MIL‐140A has been treated using a fully flexible cell, with a, b, and c as well as all three lattice angles allowed to relax independently. The resulting α and γ angles have remained close to 90°, while β has retained the characteristic monoclinic deviation from 90°. For tetragonal MIL‐163, the scaling of a and b was coupled, while c has been optimized independently and all lattice angles have been kept fixed at 90°. The same treatment of the cell degrees of freedom has been applied during the pressure‐coupled MD simulations.

For MIL‐140A and MIL‐163, the unit cells have been expanded to 1 × 1 × 2 supercells to reduce anisotropy of the lattice parameters (i.e., bringing them closer in magnitude) and to increase the number of atoms, thereby improving overall numerical stability. All MOF structures have first been equilibrated in the *NVT* ensemble at 298 K, followed by re‐equilibration and production sampling in the *NPT* ensemble at 1.013 bar, using a time step of 0.5 fs. The resulting simulation trajectories have been used to analyze unit cell parameters and volumes, and to calculate the associated X‐ray diffraction patterns. For all subsequent simulations, the time step has been increased to 2.0 fs by constraining all hydrogen‐involving bonds to their average distance using the RATTLE algorithm [87]. The systems have then been re‐equilibrated under *NVT* and *NPT* conditions for at least 50 ps prior to production sampling in the *NPT* ensemble at 298 K and 1.013 bar.

The volumetric thermal expansion coefficient $\alpha_{V}$ at temperature $T_{0}$, defined by Equation (1), describes the relative change in volume with temperature at constant pressure. The derivative has been approximated by the finite difference method given in Equation (2). Accordingly, four additional *NPT* simulations have been carried out for each system. The equidistant temperature spacing ΔT has been set to 25 K for all metal‐organic frameworks. The linear thermal expansion coefficient $\alpha_{L}$ has been calculated analogously, by replacing the volume with the respective lattice parameter.

(1) $$\alpha_{V}=\frac{1}{V_{T_{0}}}{\left(\frac{\partial V}{\partial T}\right)}_{P}$$

(2) $$\begin{array}{c} {\left(\frac{\partial V}{\partial T}\right)}_{P}\approx \frac{V_{T_{0}-2\Delta T}-8V_{T_{0}-\Delta T}+8V_{T_{0}+\Delta T}-V_{T_{0}+2\Delta T}}{12\cdot \Delta T} \end{array}$$

For the cubic metal‐organic frameworks UiO‐66, UiO‐67, and MOF‐801, the bulk modulus K has been determined from the pressure response to a small isotropic change in volume at constant temperature according to Equation (3). The corresponding derivative has been estimated using the finite difference scheme described in Equation (4). Consequently, two additional *NVT* simulations have been performed for each system at volumes ±0.1% relative to the equilibrium volume at 1.013 bar. In this context, ΔV denotes the equidistant volume difference between the reference simulation at 298 K and 1.013 bar and the two additional *NVT* simulations. The perturbed simulation cells have been generated by uniformly scaling all three lattice parameters, while keeping the angles fixed.

For the non‐cubic MOFs MIL‐140A and MIL‐163, the same procedure defines a fixed‐shape isotropic deformation path and therefore does not account for the full anisotropic elastic response. The resulting quantity is consequently denoted $K_{\mathrm{iso}}$ and referred to as the fixed‐shape isotropic volumetric modulus. For the cubic systems, this distinction does not arise and the corresponding quantity is reported as the bulk modulus K.

(3) $$\begin{array}{c} K=-V_{\mathrm{eq}}{\left(\frac{\partial P}{\partial V}\right)}_{T} \end{array}$$

(4) $$\begin{array}{c} {\left(\frac{\partial P}{\partial V}\right)}_{T}\approx \frac{P_{+0.1\%}-P_{-0.1\%}}{2\cdot \Delta V} \end{array}$$

Benchmark calculations with the HSEsol hybrid functional [70] have been carried out via the CRYSTAL23 program [88], employing the pob‐DZVP‐rev2 basis set for hydrogen, carbon, nitrogen, and oxygen [89], and the pob‐DZVP‐2018 basis set for zirconium [90]. The shrinking factor of the reciprocal lattice vectors in the Monkhorst‐Pack integration scheme [91] has been set to 8, and the self‐consistent field (SCF) energy convergence criterion to 10^−8^ Hartree. The initial structures have first been subjected to a full geometry optimization of atomic positions and lattice parameters. Subsequently, the thermal expansion coefficients of the individual MOFs have been calculated using the built‐in quasi‐harmonic approximation (QHA) module [92, 93, 94, 95] of CRYSTAL23. The corresponding bulk moduli have been computed via the equation of state module [96], based on the Birch‐Murnaghan (BMEOS) formalism [97]. The required energy minimizations with fixed cell parameters and subsequent phonon frequency calculations have been performed at equidistant volumes relative to the equilibrium volume obtained from geometry optimization. For UiO‐66, UiO‐67, MIL‐140A, and MIL‐163, four volumes spanning from 2% below to 4% above the equilibrium volume have been used, whereas for MOF‐801, 13 volumes spanning from 1.5% below to 3% above have been considered.

## 3 Results and Discussion

The results of the unit cell analysis and XRD pattern evaluation of MOF‐801, MIL‐140A, and MIL‐163 are presented in the following. In addition, the thermal expansion coefficients and volumetric stiffness measures of these frameworks, as well as of UiO‐66 and UiO‐67, are reported. For UiO‐66 and UiO‐67, unit cell analysis and XRD pattern comparisons have not been repeated in the present work, as these results have been published in our previous study [68].

Table 1 compares the lattice parameters of MOF‐801 obtained at DFTB3/3OB, MACE‐DAC‐1, and HSEsol levels of theory with literature values. DFTB3/3OB and MACE‐DAC‐1 simulation‐based results exhibit a slight overestimation compared to experimental results. This deviation may partly reflect a method‐dependent tendency to predict larger lattice parameters under the simulated conditions. In addition, differences between the idealized guest‐free frameworks considered in the simulations and the corresponding experimental samples may contribute to the observed deviations, as adsorption and residual pore species are known to affect the structural response of MOFs [101, 102]. Structures obtained by geometry optimization at DFTB3/3OB and MACE‐DAC‐1 levels of theory exhibit smaller lattice parameters than those obtained from the corresponding MD simulations, indicating positive thermal expansion behavior. Moreover, all employed methods predict a smaller lattice parameter than the DFT/PBE result reported by Chen et al. The time evolution of the lattice parameter during the DFTB3/3OB simulation and the corresponding distribution are displayed in Figure S1 and Figure S2, respectively. A comparison of the XRD pattern obtained from the DFTB3/3OB MD simulation with that reported by Chen et al. is presented in Figure 1, demonstrating excellent agreement between the two datasets.

**TABLE 1:** Comparison of the MOF‐801 lattice parameter a.

Method	a (Å)	T (K)	Source
XRD	17.8348 (17)	363	Furukawa et al. [31]
PXRD	17.910–17.930	N/A	Butova et al. [98]
MACE‐DAC‐1 OPT	17.929	0	This work
PXRD	17.9396 (7)	N/A	Vetlitsyna‐Novikova et al. [99]
MACE‐DAC‐1 MD	17.9486 (21)	298	This work
DFTB3/3OB OPT	17.999	0	This work
DFTB3/3OB MD	18.0177 (9)	298	This work
HSEsol OPT	18.05	0	This work
PBE OPT	18.2094	0	Chen et al. [100]

*Note:* For all geometry optimizations and MD simulations performed in this work, the cubic symmetry has been retained by isotropic cell scaling, preserving a = b = c and α = β = γ = 90°. DFTB3/3OB MD and MACE‐DAC‐1 MD results have been obtained from 200 ps of unconstrained MD simulations and are reported as averages over five consecutive, non‐overlapping 40 ps blocks, with standard deviations given in parentheses.

**FIGURE 1:**
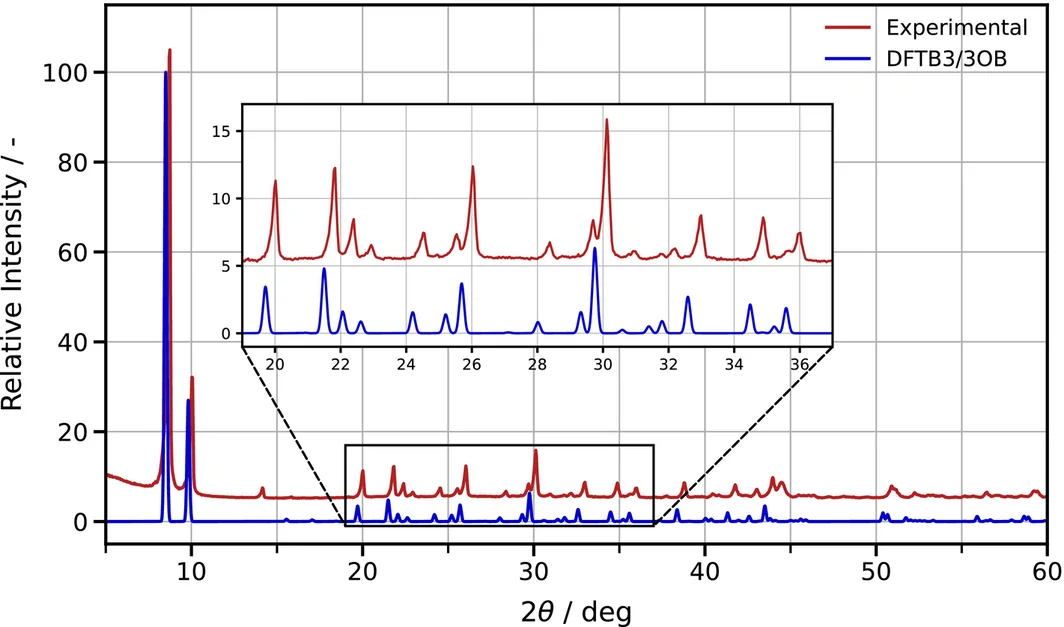
Comparison of the calculated X‐ray diffraction pattern (blue) of MOF‐801 with the experimental dataset (red) reported by Chen et al. [100] The calculated pattern has been obtained from a 200 ps unconstrained DFTB3/3OB MD simulation. A wavelength of 1.54180 Å, corresponding to Cu‐K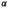 radiation, has been used for the theoretical calculation.

The lattice parameters and corresponding unit cell volumes of MIL‐140A obtained from MD simulations and geometry optimizations are summarized and compared to reported literature values in Table 2. At DFTB3/3OB level of theory, the simulated structure exhibits a slight overestimation of the unit cell volume, with a calculated value of 2120.90 (15) Å^3^ relative to the experimental reference value of 2078 Å^3^ reported by Jodłowski et al., which served as the initial configuration for our calculations. This slight overestimation may reflect both method‐dependent effects and differences between the idealized guest‐free systems and the experimental samples as previously outlined for MOF‐801. Overall, the structural parameters obtained via the DFTB3/3OB method are in good agreement with previously reported data, with lattice parameters showing small deviations and the cell volume falling within the range of values predicted by experimental measurements, DFT‐based approaches, and the MACE‐DAC‐1 MLFF. The minor deviations of the α and γ angles from exactly 90° for DFTB3/3OB and MACE‐DAC‐1 arise from the fully anisotropic treatment of the simulation cell during pressure coupling in the MD simulation and from the unconstrained optimization of the lattice parameters during energy minimization. Nevertheless, the two angles remain very close to orthogonality in all four cases, consistent with the expected monoclinic crystal system of MIL‐140A. Time evolutions of the lattice parameters and the unit cell volume computed at DFTB3/3OB level of theory are depicted in Figure S3, with the corresponding distributions shown in Figure S4. The quality of the reported results is reflected by the comparison between the computed XRD pattern and the experimental reference shown in Figure 2. The major reflections are reproduced well by the simulated dataset, indicating good overall agreement with the experiment. The most intense reflection at approximately 2θ≈6.9° is indexed to the (200) plane. In the theoretical pattern, this peak appears at slightly lower 2θ values compared to the experimental reference. This shift can be attributed to an overestimation of the lattice parameter a by the DFTB3/3OB MD simulation, which increases the corresponding interplanar spacing and consequently shifts the reflection to smaller diffraction angles according to Bragg's law.

**TABLE 2:** Comparison of the MIL‐140A lattice parameters a, b, c, α, β, γ, and unit cell volume V.

Method	a (Å)	b (Å)	c (Å)	α (°)	β (°)	γ (°)	V (Å^3^)	T (K)	Source
HSEsol OPT	25.26	11.15	7.790	90	111.4	90	2042	0	This work
PXRD	24.4243 (10)	11.1795 (10)	7.8022 (10)	90	103.861 (2)	90	2068.4 (3)	296	Guillerm et al. [35]
PXRD	25.59	11.20	7.82	90	112.5	90	2070.16	N/A	Zhang et al. [103]
PXRD	25.6368 (16)	11.2048 (7)	7.8254 (4)	90	112.417	90	2078.0 (2)	293	Jodłowski et al. [85]
B3LYP OPT	24.98	11.24	7.69	90	104.95	90	2086.9	0	Ortiz et al. [104]
MACE‐DAC‐1 MD	25.518 (14)	11.2960 (16)	7.8869 (14)	90.09 (6)	112.17 (4)	89.93 (4)	2105.4 (12)	298	This work
MACE‐DAC‐1 OPT	25.576	11.303	7.8602	90.06	111.8	89.96	2109.2	0	This work
DFTB3/3OB OPT	27.685	11.047	7.4306	89.92	111.4	90.15	2115.1	0	This work
DFTB3/3OB MD	27.766 (3)	11.0574 (11)	7.4443 (8)	90.000 (12)	111.880 (14)	89.992 (8)	2120.90 (15)	298	This work
PBE OPT	24.53	11.24	7.87	90	97.71	90	2150.4	0	Guillerm et al. [35]
B3LYP OPT	24.7627	11.2756	7.9779	90	102.482	90	2175.00	0	Ryder et al. [105]

*Note:* For the DFTB3/3OB and MACE‐DAC‐1 geometry optimizations and MD simulations performed in this work, the cell has been treated as fully flexible, allowing all six lattice parameters to relax independently. The HSEsol geometry optimization has instead been carried out under the constraints of the monoclinic space group and therefore reports α = γ = 90°. DFTB3/3OB MD and MACE‐DAC‐1 MD results have been obtained from 200 ps of unconstrained MD simulations and are reported as averages over five consecutive, non‐overlapping 40 ps blocks, with standard deviations given in parentheses.

**FIGURE 2:**
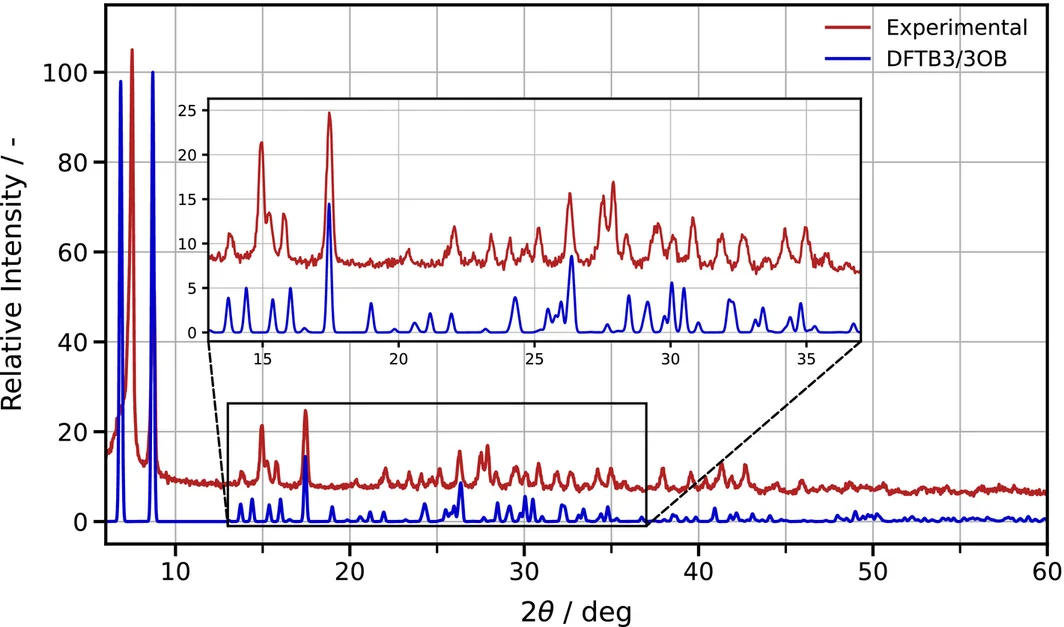
Comparison of the calculated X‐ray diffraction pattern (blue) of MIL‐140A with the experimental dataset (red) reported by Jodłowski et al. [85] The calculated pattern has been obtained from a 200 ps unconstrained DFTB3/3OB MD simulation. A wavelength of 1.54060 Å, corresponding to Cu‐K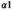 radiation, has been used for the theoretical calculation.

The two computed lattice parameters a and c of the tetragonal MIL‐163 structure, together with the associated unit cell volume V, are summarized in Table 3. Comparison with the values reported by Mouchaham et al. reveals that the MACE‐DAC‐1 model and the DFT optimization slightly underestimate the unit cell volume, whereas the DFTB3/3OB approach leads to a modest overestimation. Notably, the lattice parameter a obtained from MACE‐DAC‐1 MD simulation is in excellent agreement with the experimental reference. As discussed for the preceding MOFs, the deviation observed for DFTB3/3OB may arise from a combination of intrinsic method effects and differences between the simulated and experimental chemical environments. Structural parameters during the course of the MD simulation at DFTB3/3OB level are shown in Figure S5, while the associated distributions are presented in Figure S6. Figure 3 compares the calculated XRD pattern with that reported by Mouchaham et al. In analogy to MIL‐140A, the larger unit cell predicted by DFTB3/3OB results in a systematic shift of the reflections toward lower 2θ values. Nevertheless, the positions remain in good agreement with the experimental reference.

**TABLE 3:** Comparison of the MIL‐163 lattice parameters a and c, and unit cell volume V.

Method	a (Å)	c (Å)	V (Å^3^)	T (K)	Source
MACE‐DAC‐1 OPT	15.524	7.1297	1718.3	0	This work
MACE‐DAC‐1 MD	15.612 (7)	7.1715 (15)	1748.0 (16)	298	This work
HSEsol OPT	15.81	7.044	1760	0	This work
PXRD	15.6152 (2)	7.49628 (9)	1827.85 (4)	293	Mouchaham et al. [86]
DFTB3/3OB OPT	16.137	7.6010	1979.4	0	This work
DFTB3/3OB MD	16.167 (10)	7.595 (6)	1985.2 (28)	298	This work

*Note:* For all geometry optimizations and MD simulations performed in this work, the tetragonal symmetry has been retained by coupling the scaling of a and b, while allowing c to vary independently and keeping all lattice angles fixed at 90°. DFTB3/3OB MD and MACE‐DAC‐1 MD results have been obtained from 200 ps of unconstrained MD simulations and are reported as averages over five consecutive, non‐overlapping 40 ps blocks, with standard deviations given in parentheses.

**FIGURE 3:**
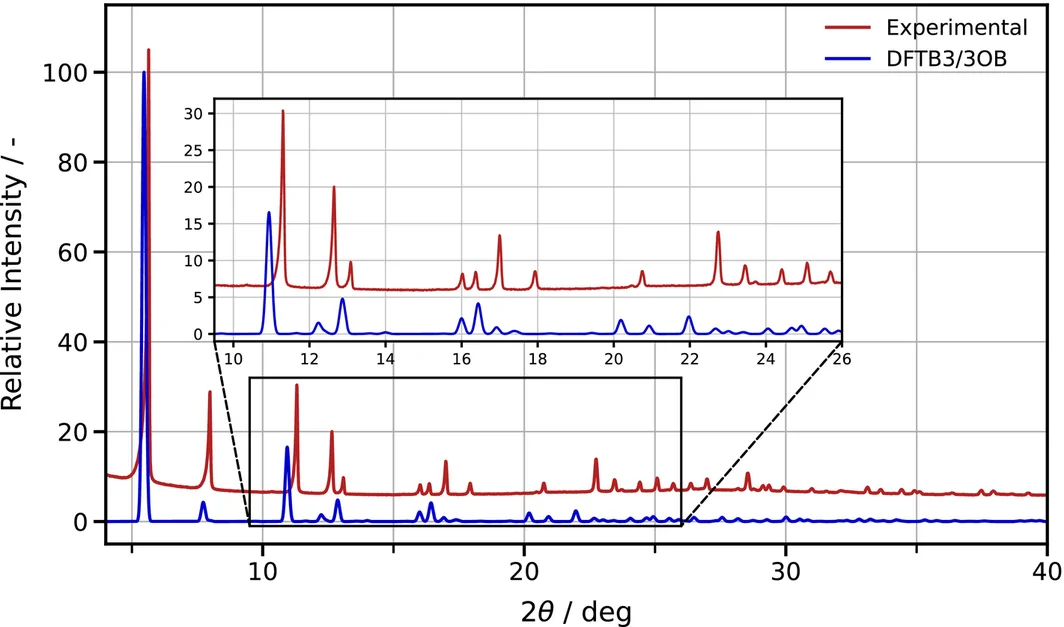
Comparison of the calculated X‐ray diffraction pattern (blue) of MIL‐163 with the experimental dataset (red) reported by Mouchaham et al. [86] The calculated pattern has been obtained from a 200 ps unconstrained DFTB3/3OB MD simulation. A wavelength of 1.54060 Å, corresponding to Cu‐K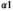 radiation, has been used for the theoretical calculation.

The thermal expansion coefficients of UiO‐66, UiO‐67, MOF‐801, MIL‐140A, and MIL‐163 at DFTB3/3OB and MACE‐DAC‐1 levels of theory have been determined from five individual *NPT* simulations per system. Simulations of the UiO MOFs have been performed for 1 ns, whereas those of MOF‐801, MIL‐140A, and MIL‐163 have been extended to 2 ns due to their smaller system sizes. Figure 4 shows the lattice parameter as a function of temperature for UiO‐66, UiO‐67, and MOF‐801, as well as the unit cell volume as a function of temperature for MIL‐140A and MIL‐163. Corresponding time evolutions of the plotted quantities and their associated distributions are given in the Figures S7–S16. Resulting thermal expansion coefficients are compared in Tables 4, 5, 6. In addition, Figure S17 shows the temperature dependence of the lattice parameters for UiO‐66, UiO‐67, and MOF‐801, and the unit cell volumes for MIL‐140A and MIL‐163, as obtained from MACE‐DAC‐1 MD simulations.

**FIGURE 4:**
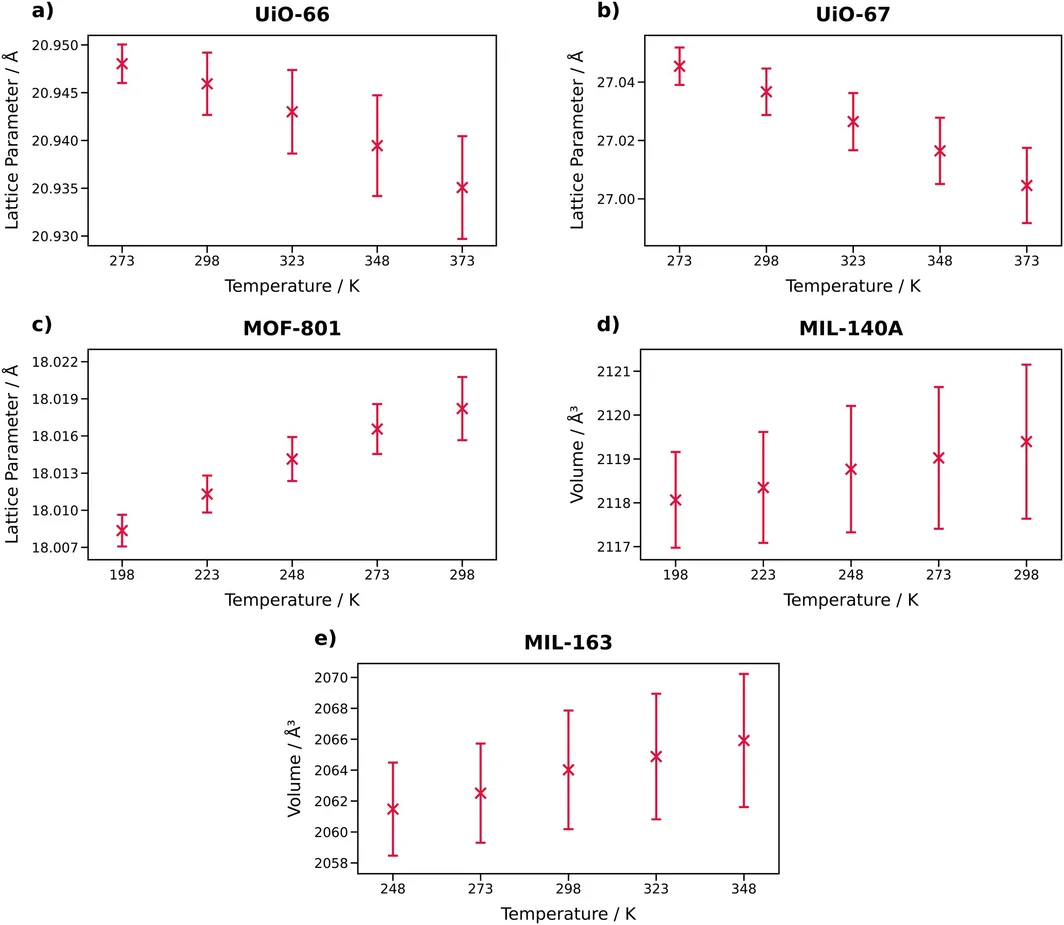
Lattice parameter as a function of temperature for (a) UiO‐66, (b) UiO‐67, and (c) MOF‐801, and unit cell volume as a function of temperature for (d) MIL‐140A and (e) MIL‐163, as determined from DFTB3/3OB MD simulations. Error bars indicate the standard deviation of the respective time‐averaged quantities.

**TABLE 4:** Comparison of the linear thermal expansion coefficients $\alpha_{L}$ of UiO‐66, UiO‐67, and MOF‐801.

MOF	Method	$\alpha_{L}$ (MK^−1^)	T (K)	Source
UiO‐66	PXRD	+4.92(81)	278–369	Burtch et al. [106]
	PXRD	−0.9	333	Vornholt et al. [107]
	BTW‐FF MD	−1.04	1–500	Bristow et al. [108]
	BTW‐FF MD	−3.1	80–500	Boyd et al. [50]
	HSEsol/QHA	−3.93	300	This work
	Custom FF MD	−5.5	280–370	Burtch et al. [106]
	PXRD	−5.55(27)	281–366	Burtch et al. [106]
	DFTB3/3OB MD	−6.19	273–373	This work
	MACE‐DAC‐1 MD	−7.63	273–373	This work
	UFF MD	−8.4	80–500	Boyd et al. [50]
	PXRD	−35.4(2)	423–523	Vornholt et al. [107]
UiO‐67	HSEsol/QHA	−1.28	300	This work
	BTW‐FF MD	−2.22	1–500	Bristow et al. [108]
	PXRD	−3.29(1)	90–420	Goodenough et al. [109]
	PXRD	−6.85(32)	279–369	Burtch et al. [106]
	Custom FF MD	−10.9	280–370	Burtch et al. [106]
	DFTB3/3OB MD	−14.9	273–373	This work
	MACE‐DAC‐1 MD	−23.2	273–373	This work
MOF‐801	DFTB3/3OB MD	+5.94	198–298	This work
	MACE‐DAC‐1 MD	+5.87	198–298	This work
	HSEsol/QHA	+3.94	300	This work

**TABLE 5:** Comparison of the MIL‐140A volumetric thermal expansion coefficient $\alpha_{V}$ and the individual linear thermal expansion coefficients $\alpha_{a}$, $\alpha_{b}$, and $\alpha_{c}$.

Method	$\alpha_{V}$ (MK^−1^)	$\alpha_{a}$ (MK^−1^)	$\alpha_{b}$ (MK^−1^)	$\alpha_{c}$ (MK^−1^)	T (K)	Source
DFTB3/3OB MD	+6.39	+2.21	+3.42	+7.84	198–298	This work
HSEsol/QHA	+6.12	+5.61	+0.344	+2.98	300	This work
MACE‐DAC‐1 MD	−8.03	−2.42	−5.96	+16.6	198–298	This work

**TABLE 6:** Comparison of the MIL‐163 volumetric thermal expansion coefficient $\alpha_{V}$ and the individual linear thermal expansion coefficients $\alpha_{a}$ and $\alpha_{c}$.

Method	$\alpha_{V}$ (MK^−1^)	$\alpha_{a}$ (MK^−1^)	$\alpha_{c}$ (MK^−1^)	T (K)	Source
MACE‐DAC‐1 MD	+35.7	+11.2	+13.4	248–348	This work
DFTB3/3OB MD	+23.4	−3.97	+31.4	248–348	This work
HSEsol/QHA	−26.1	−5.42	−15.3	300	This work

For UiO‐66 and UiO‐67, the negative linear thermal expansion coefficients predicted by DFTB3/3OB MD simulations (−6.19 and −14.9 MK^−1^, respectively) are consistent with reported literature data, with the values lying toward the stronger‐contraction end of the reported range. In comparison, MACE‐DAC‐1 MD simulations yield an even more pronounced contraction for both systems, with coefficients of −7.63 and −23.2 MK^−1^, whereas HSEsol/QHA calculations yield a weaker contraction, with values of −3.93 and −1.28 MK^−1^. For MOF‐801, all three methods applied in this study consistently predict positive linear thermal expansion coefficients, with values that are in excellent agreement, namely +5.94 (DFTB3/3OB), +5.87 (MACE‐DAC‐1), and +3.94 MK^−1^ (HSEsol/QHA). In the case of MIL‐140A, the two simulation‐based approaches produce qualitatively different trends. DFTB3/3OB yields a positive volumetric thermal expansion coefficient of +6.39 MK^−1^, whereas MACE‐DAC‐1 gives a negative value of −8.03 MK^−1^. The linear thermal expansion coefficients along the a and b directions display the same discrepancy. Only along the c direction are both coefficients positive, although they still differ significantly. However, the DFTB3/3OB results are in good qualitative agreement with the HSEsol/QHA predictions, with an almost perfect match to the HSEsol/QHA volumetric thermal expansion coefficient of +6.12 MK^−1^. Figure S18 shows the temperature dependence of the lattice parameters of MIL‐140A as obtained from DFTB3/3OB MD simulations. Examining MIL‐163, the volumetric thermal expansion coefficients calculated from DFTB3/3OB (+23.4 MK^−1^) and MACE‐DAC‐1 (+35.7 MK^−1^) are both positive, with the latter indicating a noticeably stronger volumetric thermal expansion. However, the underlying contributions to these values differ significantly between the two methods. While DFTB3/3OB predicts contraction along the a and b lattice directions—consistent with HSEsol/QHA—MACE‐DAC‐1 instead predicts expansion along these axes. Along the c direction, both methods yield positive thermal expansion coefficients, although the magnitudes differ substantially. In contrast, the HSEsol/QHA calculation yields negative thermal expansion along all lattice directions, resulting in an overall negative volumetric thermal expansion coefficient of −26.1 MK^−1^. The time‐averaged a and c lattice parameters of MIL‐163 at the investigated temperatures, as determined from DFTB3/3OB MD simulations, are presented in Figure S19. Notably, the positive and negative thermal expansion trends predicted by DFTB3/3OB and MACE‐DAC‐1 for MOF‐801, MIL‐140A, and MIL‐163, as reported in Tables 4, 5, 6, are consistently reflected in the corresponding changes in lattice parameters between the geometry‐optimized structures and the MD simulations presented in Tables 1, 2, 3.

To determine the bulk moduli K of the cubic MOFs and the fixed‐shape isotropic volumetric moduli $K_{\mathrm{iso}}$ of the non‐cubic frameworks via DFTB3/3OB and MACE‐DAC‐1, two additional *NVT* simulations per system have been carried out. Simulations of UiO‐66 and UiO‐67 have been carried out for 1 ns, while trajectories of MOF‐801 and the MIL‐type MOFs have been extended to 2 ns owing to the smaller number of atoms in their simulation boxes. Results of the bulk modulus calculations of the cubic MOFs are listed in Table 7, whereas results for the non‐cubic MIL‐140A and MIL‐163 are summarized in Table 8. As MACE‐DAC‐1 has not been specifically validated for elastic properties, the corresponding results are considered here as comparative MLFF predictions rather than as reference values. The time evolutions of the pressure from the additional DFTB3/3OB *NVT* simulations performed at volumes 0.1% above and below the average volume at 298 K and 1.013 bar, together with the corresponding pressure distributions, are shown in the Figures S20–S29.

**TABLE 7:** Comparison of the bulk moduli K of UiO‐66, UiO‐67, and MOF‐801.

MOF	Method	K (GPa)	Source
UiO‐66	VMOF‐FF/VRH	19.0	Bristow et al. [110]
	MACE‐DAC‐1 MD	20.1 (14)	This work
	Custom FF MD	22.2	Rogge et al. [111]
	BTW‐FF/MEOS	27.1	Boyd et al. [50]
	BTW‐FF/BMEOS	27.15	Bristow et al. [108]
	DP/BMEOS	31.88	Achar et al. [112]
	PBE/BMEOS	32.11	Achar et al. [112]
	DFTB3/3OB MD	32.2 (25)	This work
	PBE/BMEOS	36.62	Yang et al. [113]
	PXRD/BMEOS	37.9 (6)	Redfern et al. [114]
	PBEsol/BMEOS	40.4	Bristow et al. [110]
	PBE/MEOS	41.01	Wu et al. [115]
	UFF/MEOS	42.4	Boyd et al. [50]
	HSEsol/BMEOS	43.0	This work
UiO‐67	MACE‐DAC‐1 MD	4.6 (12)	This work
	VMOF‐FF/VRH	11.7	Bristow et al. [110]
	Custom FF MD	13.3	Rogge et al. [111]
	PBE/MEOS	17.15	Wu et al. [115]
	DFTB3/3OB MD	18.9 (29)	This work
	BTW‐FF/BMEOS	19.15	Bristow et al. [108]
	PXRD/BMEOS	21.1 (6)	Redfern et al. [114]
	PBEsol/BMEOS	21.9	Bristow et al. [110]
	PBE/BMEOS	22.10	Yang et al. [113]
	HSEsol/BMEOS	22.2	This work
MOF‐801	MACE‐DAC‐1 MD	21.9 (12)	This work
	DFTB3/3OB MD	31 (4)	This work
	HSEsol/BMEOS	45.2	This work

*Note:* Values are derived from the Voigt–Reuss–Hill (VRH) average, the Murnaghan equation of state (MEOS), the Birch–Murnaghan equation of state (BMEOS), or molecular dynamics (MD) simulations. For the DFTB3/3OB MD and MACE‐DAC‐1 MD simulations, the second halves of the pressure trajectories have been divided into five non‐overlapping blocks of equal length. The bulk moduli have then been determined for each block, and average values over the five blocks are reported, with standard deviations given in parentheses.

**TABLE 8:** Comparison of the conventional bulk moduli K and fixed‐shape isotropic volumetric moduli $K_{\mathrm{iso}}$ of the non‐cubic frameworks MIL‐140A and MIL‐163.

MOF	Method	K or $K_{\mathrm{iso}}$ (GPa)	Source
MIL‐140A	HSEsol/BMEOS	25.8	This work
	MACE‐DAC‐1 MD	30 (3)	This work
	B3LYP/VRH	45.28	Ryder et al. [105]
	DFTB3/3OB MD	47 (4)	This work
MIL‐163	DFTB3/3OB MD	8.9 (15)	This work
	MACE‐DAC‐1 MD	11 (4)	This work
	HSEsol/BMEOS	28.0	This work

*Note:* Here, K denotes the bulk modulus obtained from either the Voigt–Reuss–Hill (VRH) average or the Birch–Murnaghan equation of state (BMEOS), whereas $K_{\mathrm{iso}}$ denotes the fixed‐shape isotropic volumetric modulus obtained from molecular dynamics (MD) simulations. For the DFTB3/3OB MD and MACE‐DAC‐1 MD simulations, the second halves of the pressure trajectories have been divided into five non‐overlapping blocks of equal length. $K_{\mathrm{iso}}$ has then been determined for each block, and average values over the five blocks are reported, with standard deviations given in parentheses.

The bulk modulus of UiO‐66 of 32.2(25) GPa calculated using DFTB3/3OB is in excellent agreement with the reference computed at PBE/BMEOS level of theory by Achar et al. [112] (32.11 GPa). Moreover, it lies near the midpoint of the reported literature range, which spans from 19.0 to 42.4 GPa. A similar trend is observed for UiO‐67, for which a bulk modulus of 18.9(29) GPa is obtained, in close agreement with the value of 19.15 GPa predicted by Bristow et al. [108] using the BTW force field and fitting to the Birch‐Murnaghan equation of state. In contrast, the MACE‐DAC‐1 model predicts significantly lower bulk moduli for both UiO frameworks, yielding values of 20.1(14) and 4.6(12) GPa for UiO‐66 and UiO‐67, respectively, while HSEsol/BMEOS yields the highest bulk moduli of 43.0 GPa for UiO‐66 and 22.2 GPa for UiO‐67. This trend is further observed for MOF‐801, for which bulk moduli of 21.9(12), 31(4), and 45.2 GPa have been obtained at MACE‐DAC‐1, DFTB3/3OB, and HSEsol/BMEOS level of theory, respectively.

For the non‐cubic MIL‐140A and MIL‐163 frameworks, the MD‐derived quantities correspond to the fixed‐shape isotropic volumetric modulus $K_{\mathrm{iso}}$, as the volume perturbations have been generated by uniformly scaling the lattice parameters while retaining the equilibrium cell shape. These values are therefore directly comparable between DFTB3/3OB and MACE‐DAC‐1, since both approaches employ the same deformation protocol, but are not strictly equivalent to the bulk moduli K obtained from the HSEsol/BMEOS calculations or from the Voigt‐Reuss‐Hill averages reported in the literature. In case of MIL‐140A, DFTB3/3OB and MACE‐DAC‐1 yield $K_{\mathrm{iso}}$ values of 47(4) and 30(3) GPa, respectively. For reference, the corresponding conventional bulk moduli amount to 25.8 GPa at HSEsol/BMEOS level of theory and 45.28 GPa in the B3LYP/VRH calculations of Ryder et al. [105] Although the DFTB3/3OB value is numerically close to the B3LYP/VRH result, this agreement should be interpreted with caution since the two values characterize different mechanical responses.

For MIL‐163, the two MD‐based approaches yield comparable fixed‐shape isotropic volumetric moduli of 8.9(15) GPa for DFTB3/3OB and 11(4) GPa for MACE‐DAC‐1. The HSEsol/BMEOS calculation, in contrast, gives a conventional bulk modulus of 28.0 GPa. As for MIL‐140A, the difference between MD‐derived $K_{\mathrm{iso}}$ values and the HSEsol bulk modulus cannot be attributed solely to differences between the underlying computational methods, since the quantities are defined using different deformation constraints.

## 4 Conclusion

In this work, the recently introduced extension of the 3OB parameter set [72] for zirconium [68] has been benchmarked for a set of Zr‐based metal‐organic frameworks that span different secondary building units and topologies, namely UiO‐66, UiO‐67, MOF‐801, MIL‐140A, and MIL‐163. Thermomechanical properties, including thermal expansion coefficients as well as bulk moduli for the cubic frameworks and fixed‐shape isotropic volumetric moduli for the non‐cubic frameworks, have been obtained from molecular dynamics simulations and evaluated against available literature data. In addition, lattice parameters and X‐ray diffraction (XRD) patterns have been computed for MOF‐801, MIL‐140A, and MIL‐163 and compared to reported experimental reference patterns. All results have been further contextualized through an independent comparison with MACE‐DAC‐1 MLFF predictions, while hybrid DFT (HSEsol) calculations have been employed as an additional high‐level reference.

Overall, DFTB3/3OB provides a consistent structural description across the studied frameworks. The modest positive deviations in the calculated lattice parameters may originate from a combination of limitations inherent to the method and differences between the simulated empty‐pore state and potentially guest‐containing experimental structures. Importantly, these deviations are small enough that the dominant reflections of the experimental XRD patterns are accurately reproduced. Notably, MACE‐DAC‐1 consistently yields smaller unit cell volumes than DFTB3/3OB for all investigated MOFs. Regarding the thermal response, DFTB3/3OB captures the experimentally observed negative thermal expansion (NTE) of the UiO frameworks and predicts positive thermal expansion for MOF‐801. For the MIL‐type systems, DFTB3/3OB yields positive volumetric expansion for MIL‐140A and MIL‐163, with qualitative agreement to HSEsol/QHA for MIL‐140A. At the same time, the pronounced method dependence found for MIL‐140A and especially MIL‐163 highlights that thermal expansion in these anisotropic frameworks is sensitive to the underlying potential energy surface and requires further systematic validation.

The bulk moduli obtained at DFTB3/3OB level of theory are in line with the reported reference ranges for UiO‐66 and UiO‐67. For these systems, DFTB3/3OB predicts intermediate stiffness, compared to the softer MACE‐DAC‐1 predictions and the stiffer HSEsol/BMEOS reference values. The same ordering is observed in case of MOF‐801, indicating a consistent trend across the cubic subset of the benchmark systems. For the non‐cubic MIL‐140A and MIL‐163, the MD simulations instead yield fixed‐shape isotropic volumetric moduli, which permit direct comparison between DFTB3/3OB and MACE‐DAC‐1 but are not strictly equivalent to the conventional bulk moduli. $K_{\mathrm{iso}}$ determined from DFTB3/3OB is numerically close to the literature reference provided by Ryder et al.; however, given the different deformation definitions, this similarity should be regarded as qualitative rather than as a direct quantitative validation.

Taken together, these results support DFTB3/3OB as an efficient electronic‐structure‐based approach for predictive simulations of zirconium‐based MOFs, including frameworks beyond the archetypal UiO family. Future work will focus on the investigation and validation of guest@MOF hybrid systems.

## Funding

This work was supported by Austrian Science Fund (I6735, P35427).

## Conflicts of Interest

The authors declare no conflicts of interest.

## Supporting information


**Figure S1:** Time evolution of the lattice parameter a of MOF‐801. The simulation trajectory has been obtained at DFTB3/3OB level of theory at 298 K and 1.013 bar.
**Figure S2:** Distribution of the lattice parameter a of MOF‐801 corresponding to the 200 ps DFTB3/3OB trajectory shown in Figure S1. The histogram has been constructed from the trajectory obtained at 298 K and 1.013 bar using 50 bins.
**Figure S3:** Time evolution of the lattice parameters a, b, c, α, β, γ, and the unit cell volume of MIL‐140A. The simulation trajectory has been obtained at DFTB3/3OB level of theory at 298 K and 1.013 bar.
**Figure S4:** Distribution of the lattice parameters a, b, c, α, β, γ, and the unit cell volume of MIL‐140A corresponding to the 200 ps DFTB3/3OB trajectory shown in Figure S3. The histograms have been constructed from the trajectory obtained at 298 K and 1.013 bar using 50 bins each.
**Figure S5:** Time evolution of the lattice parameters a and c, and the unit cell volume of MIL‐163. The simulation trajectory has been obtained at DFTB3/3OB level of theory at 298 K and 1.013 bar.
**Figure S6:** Distribution of the lattice parameters a and c, and the unit cell volume of MIL‐163 corresponding to the 200 ps DFTB3/3OB trajectory shown in Figure S5. The histograms have been constructed from the trajectory obtained at 298 K and 1.013 bar using 50 bins each.
**Figure S7:** Time evolution of the lattice parameter of UiO‐66 at five equidistant temperature points in the range from 273 to 373 K. The MD simulations have been carried out at DFTB3/3OB level of theory. Associated averages are indicated by a dashed black line.
**Figure S8:** Distribution of the lattice parameter of UiO‐66 at five equidistant temperature points in the range from 273 to 373 K, corresponding to the trajectories shown in Figure S7. The histograms have been constructed from the DFTB3/3OB MD trajectories using 50 bins each.
**Figure S9:** Time evolution of the lattice parameter of UiO‐67 at five equidistant temperature points in the range from 273 to 373 K. The MD simulations have been carried out at DFTB3/3OB level of theory. Associated averages are indicated by a dashed black line.
**Figure S10:** Distribution of the lattice parameter of UiO‐67 at five equidistant temperature points in the range from 273 to 373 K, corresponding to the trajectories shown in Figure S9. The histograms have been constructed from the DFTB3/3OB MD trajectories using 50 bins each.
**Figure S11:** Time evolution of the lattice parameter of MOF‐801 at five equidistant temperature points in the range from 198 to 298 K. The MD simulations have been carried out at DFTB3/3OB level of theory. Associated averages are indicated by a dashed black line.
**Figure S12:** Distribution of the lattice parameter of MOF‐801 at five equidistant temperature points in the range from 198 to 298 K, corresponding to the trajectories shown in Figure S11. The histograms have been constructed from the DFTB3/3OB MD trajectories using 50 bins each.
**Figure S13:** Time evolution of the unit cell volume of MIL‐140A at five equidistant temperature points in the range from 198 to 298 K. The MD simulations have been carried out at DFTB3/3OB level of theory. Associated averages are indicated by a dashed black line.
**Figure S14:** Distribution of the unit cell volume of MIL‐140A at five equidistant temperature points in the range from 198 to 298 K, corresponding to the trajectories shown in Figure S13. The histograms have been constructed from the DFTB3/3OB MD trajectories using 50 bins each.
**Figure S15:** Time evolution of the unit cell volume of MIL‐163 at five equidistant temperature points in the range from 248 to 348 K. The MD simulations have been carried out at DFTB3/3OB level of theory. Associated averages are indicated by a dashed black line.
**Figure S16:** Distribution of the unit cell volume of MIL‐163 at five equidistant temperature points in the range from 248 to 348 K, corresponding to the trajectories shown in Figure S15. The histograms have been constructed from the DFTB3/3OB MD trajectories using 50 bins each.
**Figure S17:** Lattice parameter as a function of temperature for (a) UiO‐66, (b) UiO‐67, and (c) MOF‐801, and unit cell volume as a function of temperature for (d) MIL‐140A and (e) MIL‐163, as determined from MACE‐DAC‐1 MD simulations.
**Figure S18:** Temperature dependence of the lattice parameters a, b, c, α, β, and γ of MIL140A, as determined from five independent 2 ns DFTB3/3OB MD simulations. Error bars indicate the standard deviation of the respective time‐averaged quantities.
**Figure S19:** Temperature dependence of the lattice parameters a and c of MIL‐163, as determined from five independent 2 ns DFTB3/3OB MD simulations. Error bars indicate the standard deviation of the respective time‐averaged quantities.
**Figure S20:** Time evolution of the pressure during the two NVT simulations of UiO‐66 at DFTB3/3OB level of theory at volumes 0.1% above a) and below b) the average volume at 298 K and 1.013 bar. The blue lines represent the associated running average over a window size of 0.01 ns. The dashed vertical lines at 0.5 ns mark the starting point for calculating the average pressures of −375 and 268 bar, indicated by the orange lines.
**Figure S21:** Distribution of the pressure during the two NVT simulations of UiO‐66 at DFTB3/3OB level of theory, corresponding to the trajectories shown in Figure S20. The simulations have been performed at volumes 0.1% above (a) and below (b) the average volume at 298 K and 1.013 bar. The histograms have been constructed using 50 bins each.
**Figure S22:** Time evolution of the pressure during the two NVT simulations of UiO‐67 at DFTB3/3OB level of theory at volumes 0.1% above (a) and below (b) the average volume at 298 K and 1.013 bar. The blue lines represent the associated running average over a window size of 0.01 ns. The dashed vertical lines at 0.5 ns mark the starting point for calculating the average pressures of −256 and 122 bar, indicated by the orange lines.
**Figure S23:** Distribution of the pressure during the two NVT simulations of UiO‐67 at DFTB3/3OB level of theory, corresponding to the trajectories shown in Figure S22. The simulations have been performed at volumes 0.1% above (a) and below (b) the average volume at 298 K and 1.013 bar. The histograms have been constructed using 50 bins each.
**Figure S24:** Time evolution of the pressure during the two simulations of MOF‐801 at DFTB3/3OB level of theory at volumes 0.1% above (a) and below (b) the average volume at 298 K and 1.013 bar. The blue lines represent the associated running average over a window size of 0.02 ns. The dashed vertical lines at 1.0 ns mark the starting point for calculating the average pressures of −291 and 336 bar, indicated by the orange lines.
**Figure S25:** Distribution of the pressure during the two NVT simulations of MOF‐801 at DFTB3/3OB level of theory, corresponding to the trajectories shown in Figure S24. The simulations have been performed at volumes 0.1% above (a) and below (b) the average volume at 298 K and 1.013 bar. The histograms have been constructed using 50 bins each.
**Figure S26:** Time evolution of the pressure during the two simulations of MIL‐140A at DFTB3/3OB level of theory at volumes 0.1% above (a) and below (b) the average volume at 298 K and 1.013 bar. The blue lines represent the associated running average over a window size of 0.02 ns. The dashed vertical lines at 1.0 ns mark the starting point for calculating the average pressures of −690 and 240 bar, indicated by the orange lines.
**Figure S27:** Distribution of the pressure during the two NVT simulations of MIL‐140A at DFTB3/3OB level of theory, corresponding to the trajectories shown in Figure S26. The simulations have been performed at volumes 0.1% above (a) and below (b) the average volume at 298 K and 1.013 bar. The histograms have been constructed using 50 bins each.
**Figure S28:** Time evolution of the pressure during the two NVT simulations of MIL‐163 at DFTB3/3OB level of theory at volumes 0.1% above (a) and below (b) the average volume at 298 K and 1.013 bar. The blue lines represent the associated running average over a window size of 0.02 ns. The dashed vertical lines at 1.0 ns mark the starting point for calculating the average pressures of −97 and 81 bar, indicated by the orange lines.
**Figure S29:** Distribution of the pressure during the two NVT simulations of MIL‐163 at DFTB3/3OB level of theory, corresponding to the trajectories shown in Figure S28. The simulations have been performed at volumes 0.1% above (a) and below (b) the average volume at 298 K and 1.013 bar. The histograms have been constructed using 50 bins each.
**Supporting Information:** Time evolutions and histograms of lattice parameters and unit cell volumes from DFTB3/3OB molecular dynamics simulations for MOF‐801, MIL‐140A, and MIL‐163; time evolutions and histograms of the lattice parameters for UiO‐66 and UiO‐67 and of the unit cell volumes for MOF‐801, MIL‐140A, and MIL‐163 at the individual temperature points used for the thermal expansion analysis at DFTB3/3OB level of theory; temperature dependence of lattice parameters and unit cell volumes obtained from MACE‐DAC‐1 MD simulations; temperature dependence of MIL‐140A and MIL‐163 lattice parameters for DFTB3/3OB MD simulations; time evolutions and histograms of the pressure in the additional DFTB3/3OB NVT simulations used to compute bulk moduli for the cubic frameworks and fixed‐shape isotropic volumetric moduli for the non‐cubic frameworks.

## Data Availability

The data that support the findings of this study are available from the corresponding author upon reasonable request.
