# Supplementary material for: Assessing Structural, Thermal, and Mechanical Properties of Zirconium‐Based Metal‐Organic Frameworks via DFTB3/3OB MD Simulations: A Benchmark Beyond UiO Frameworks
Source: J Comput Chem. 2026 Oct 28;47(28):e70536. doi: 10.1002/jcc.70536 (PMC13717057; doi:10.1002/jcc.70536)
Supplement: Supplementary file 1 [file JCC-47-0-s001.pdf]

Supporting Information:

Assessing Structural, Thermal, and Mechanical Properties of Zirconium-Based Metal-Organic Frameworks via DFTB3/3OB MD Simulations: A Benchmark Beyond UiO Frameworks

Armin Penz and Thomas S. Hofer*

Department of General, Inorganic and Theoretical Chemistry, University of Innsbruck

E-mail: T.Hofer@uibk.ac.at

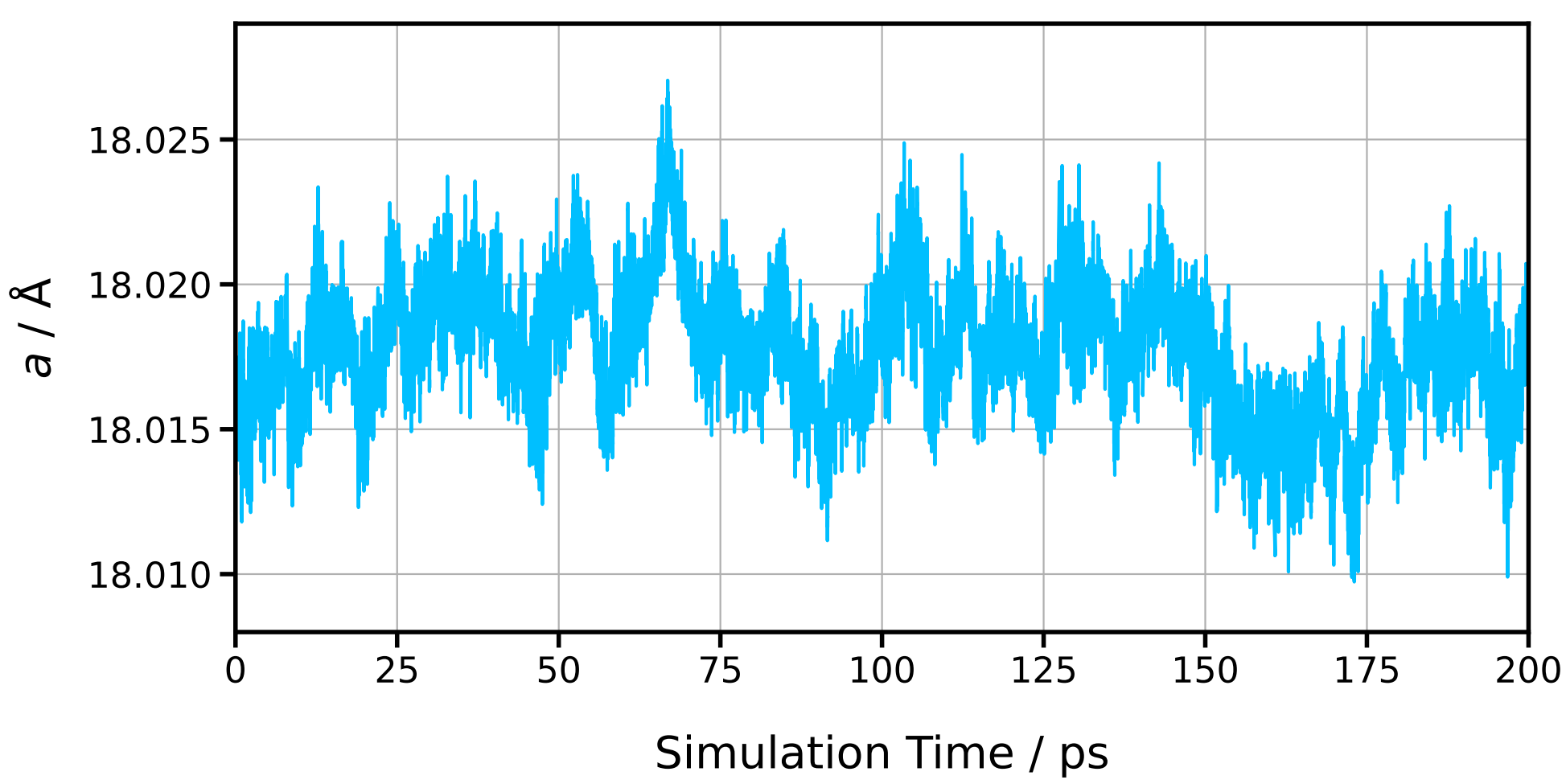

Figure S1: Time evolution of the lattice parameter a of MOF-801. The simulation trajectory has been obtained at DFTB3/3OB level of theory at 298 K and 1.013 bar.

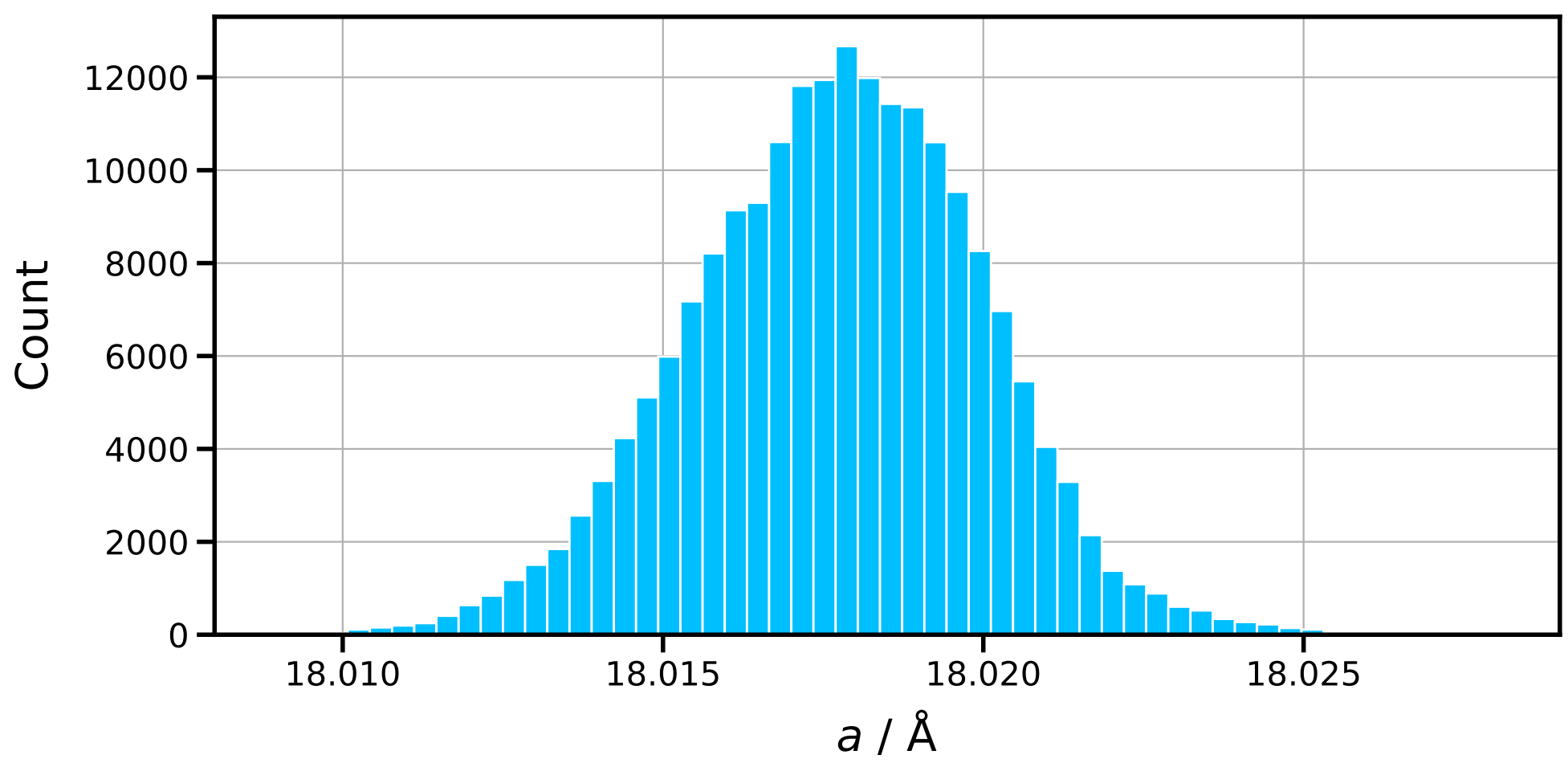

Figure S2: Distribution of the lattice parameter a of MOF-801 corresponding to the 200 ps DFTB3/3OB trajectory shown in Figure S1. The histogram has been constructed from the trajectory obtained at 298 K and 1.013 bar using 50 bins.

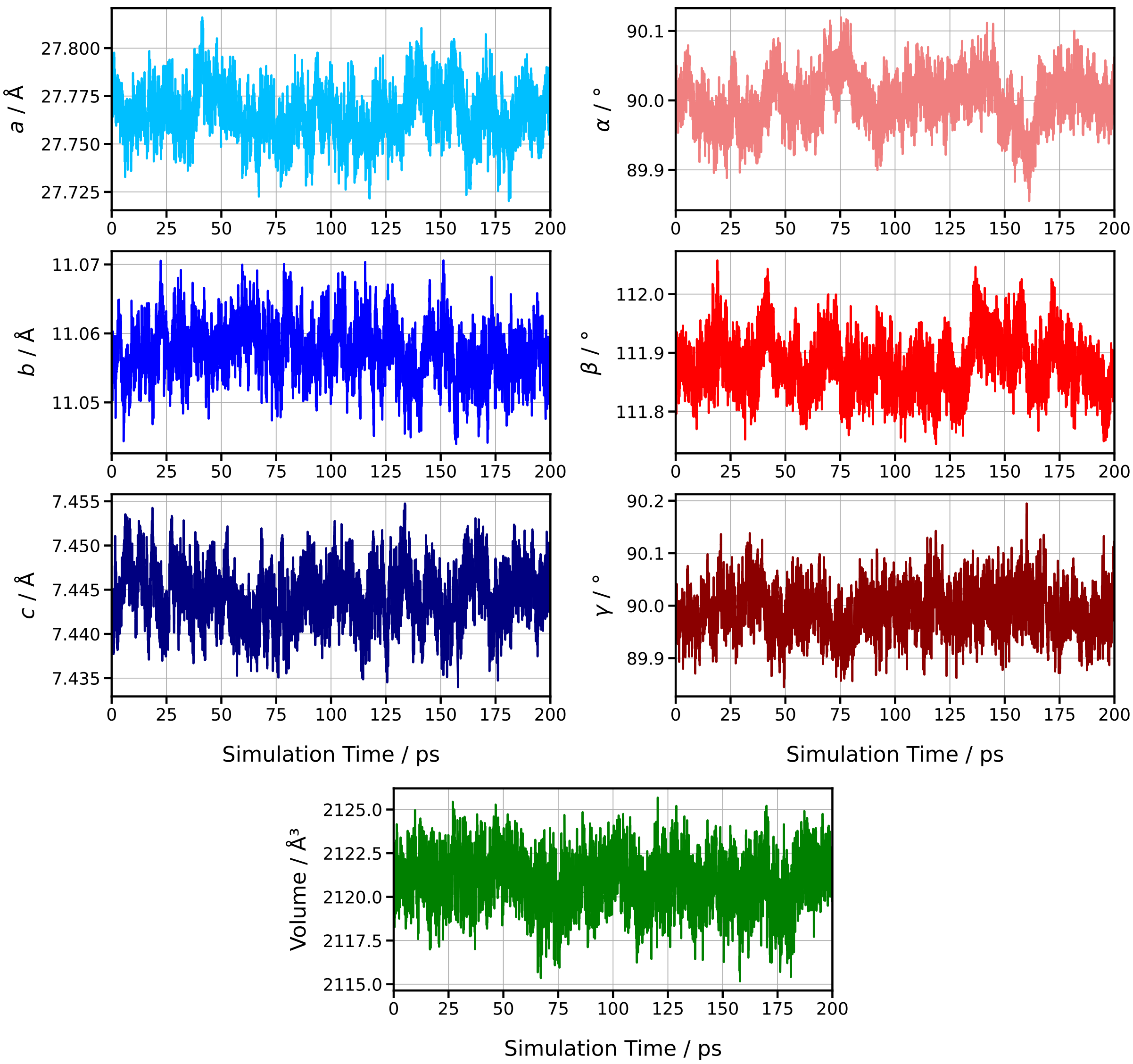

Figure S3: Time evolution of the lattice parameters a, b, c, α, β, γ, and the unit cell volume of MIL-140A. The simulation trajectory has been obtained at DFTB3/3OB level of theory at 298 K and 1.013 bar.

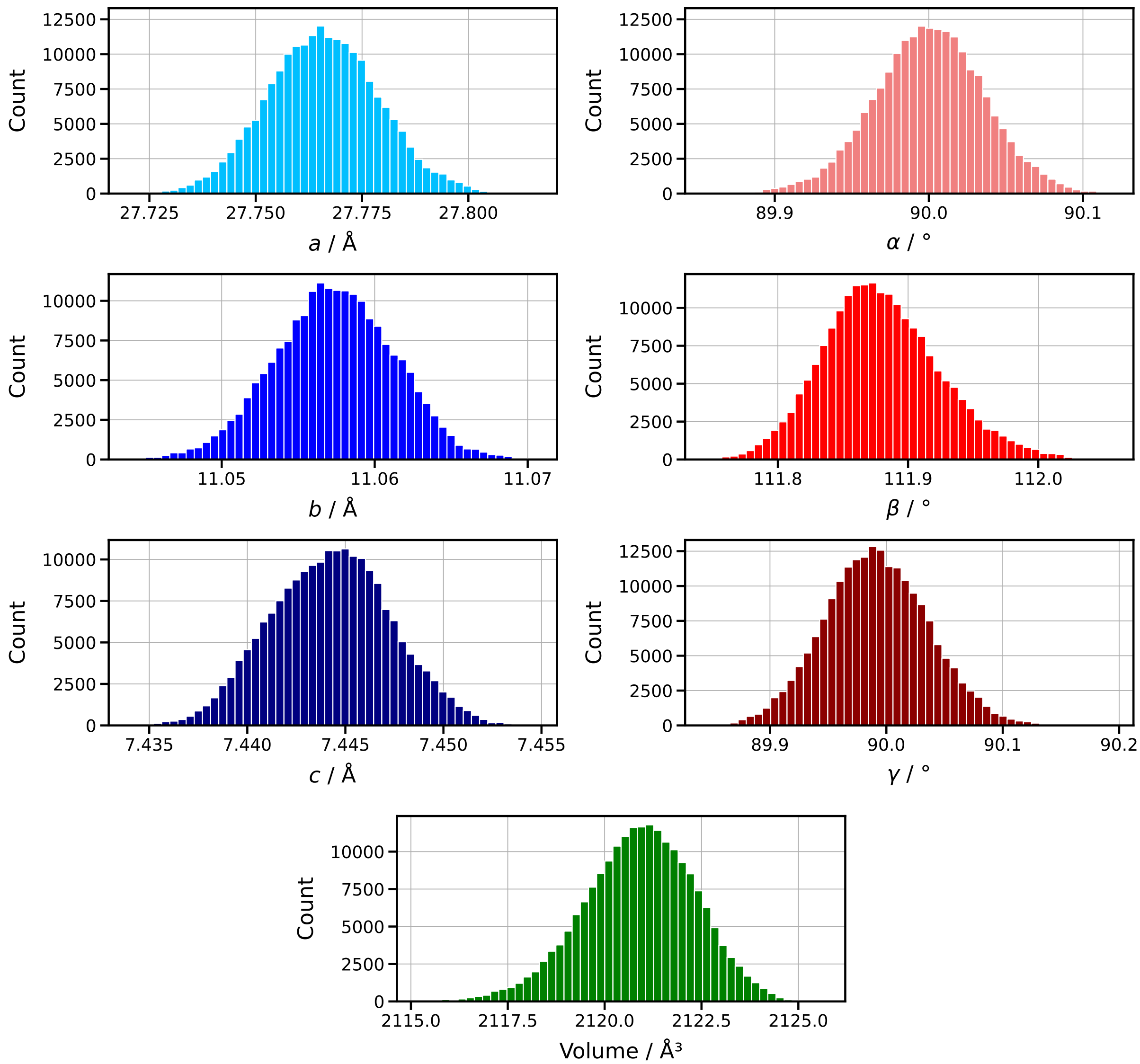

Figure S4: Distribution of the lattice parameters a, b, c, α, β, γ, and the unit cell volume of MIL-140A corresponding to the 200 ps DFTB3/3OB trajectory shown in Figure S3. The histograms have been constructed from the trajectory obtained at 298 K and 1.013 bar using 50 bins each.

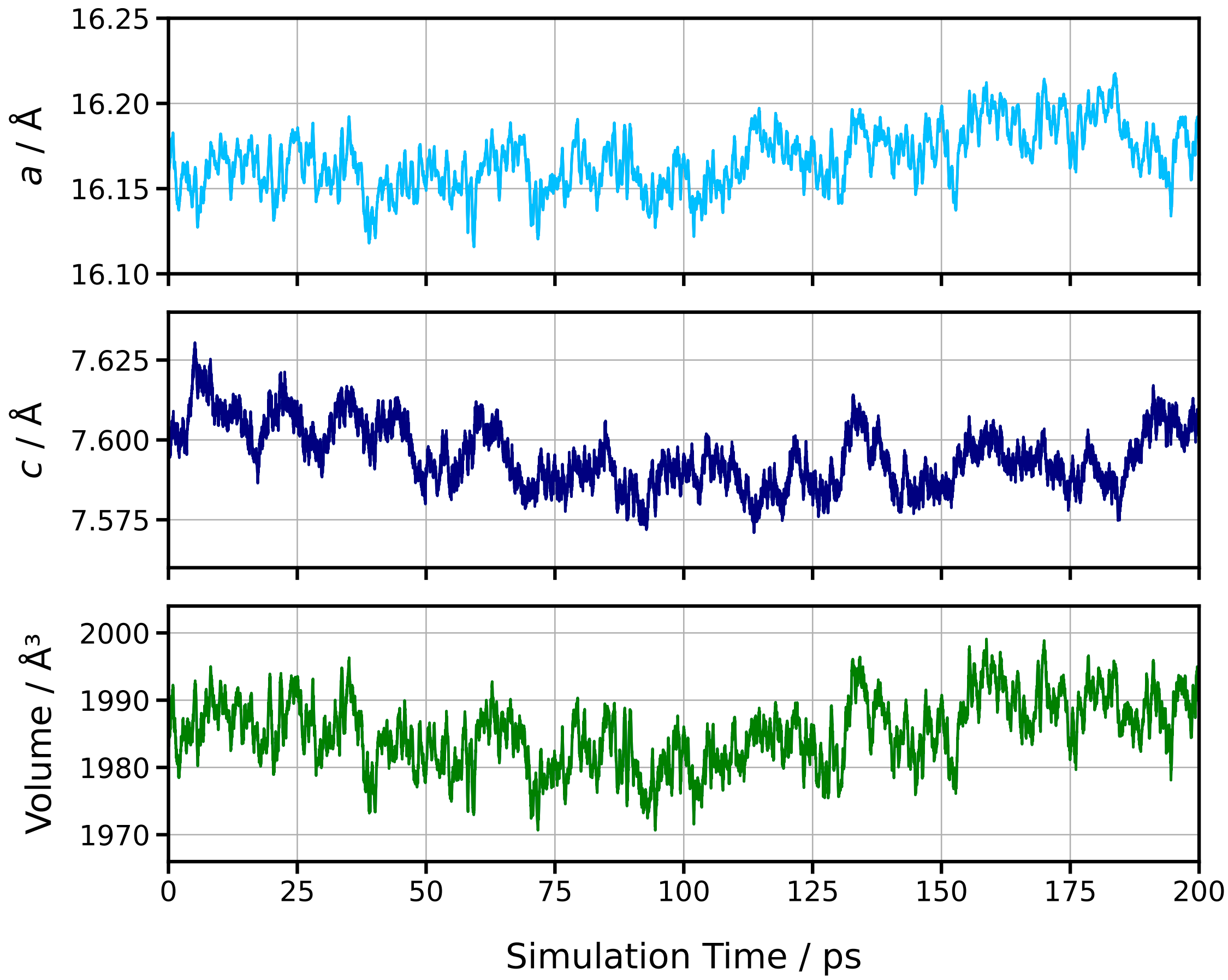

Figure S5: Time evolution of the lattice parameters a and c, and the unit cell volume of MIL-163. The simulation trajectory has been obtained at DFTB3/3OB level of theory at 298 K and 1.013 bar.

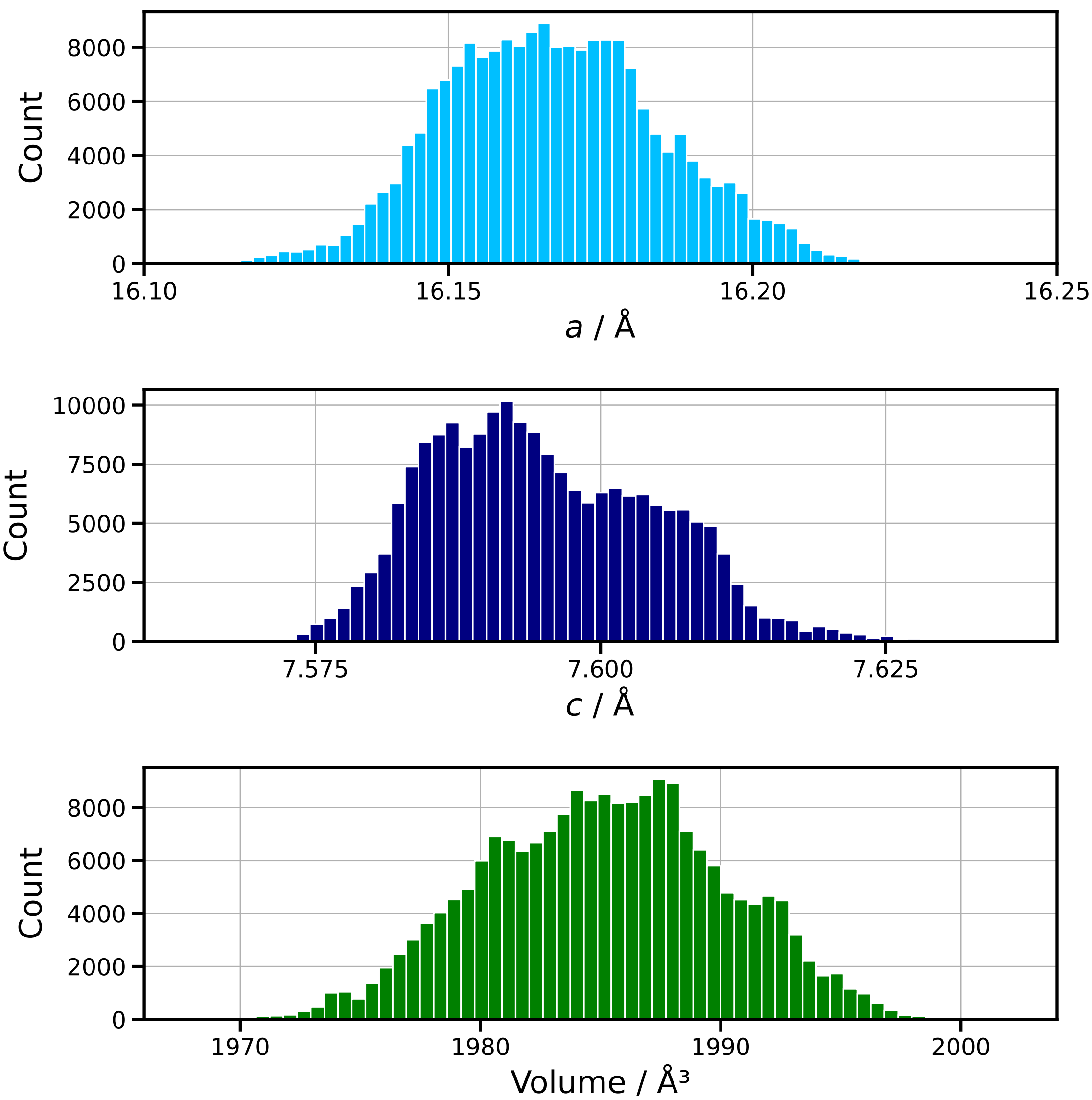

Figure S6: Distribution of the lattice parameters a and c, and the unit cell volume of MIL-163 corresponding to the 200 ps DFTB3/3OB trajectory shown in Figure S5. The histograms have been constructed from the trajectory obtained at 298 K and 1.013 bar using 50 bins each.

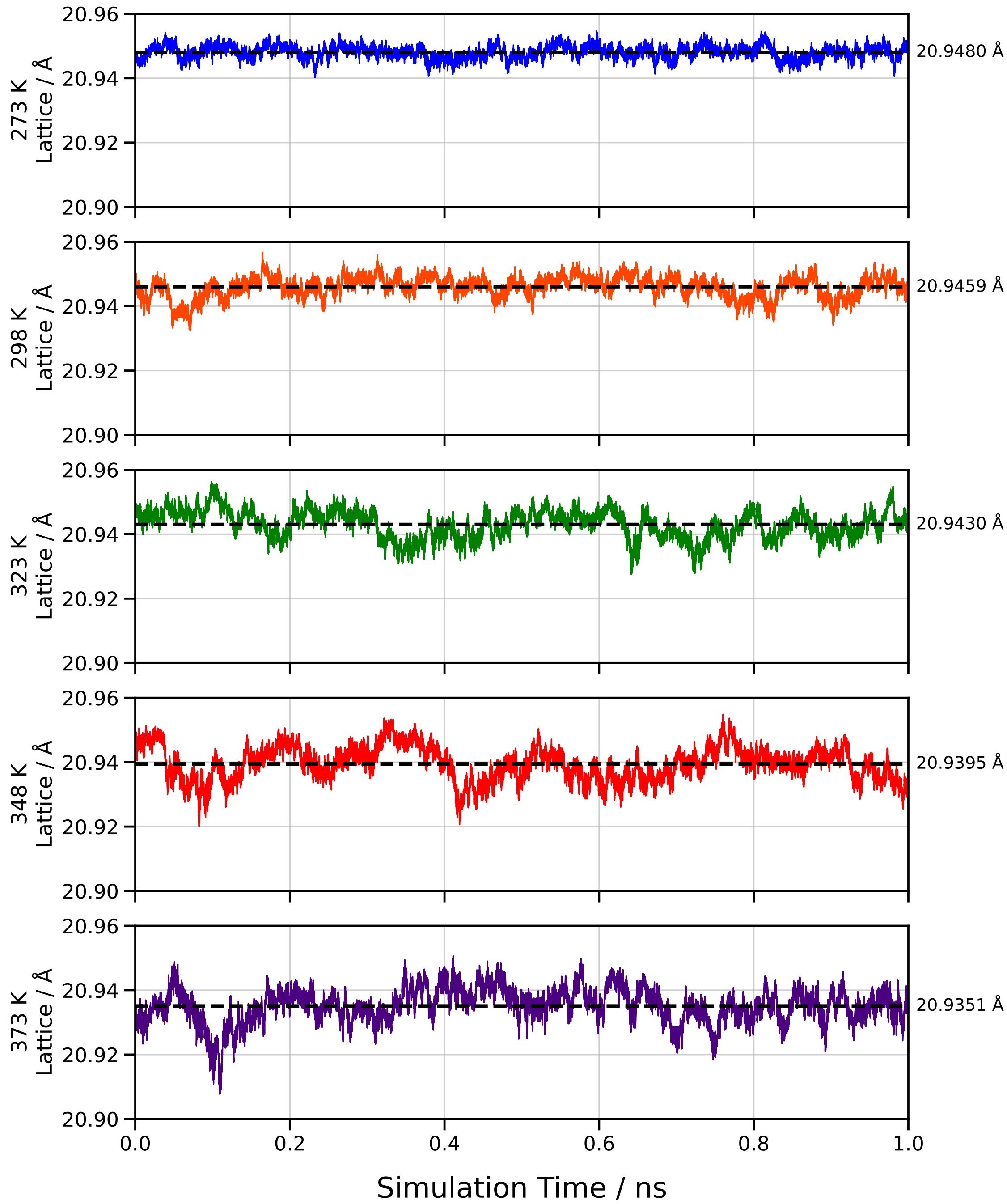

Figure S7: Time evolution of the lattice parameter of UiO-66 at five equidistant temperature points in the range from 273 to 373 K. The MD simulations have been carried out at DFTB3/3OB level of theory. Associated averages are indicated by a dashed black line.

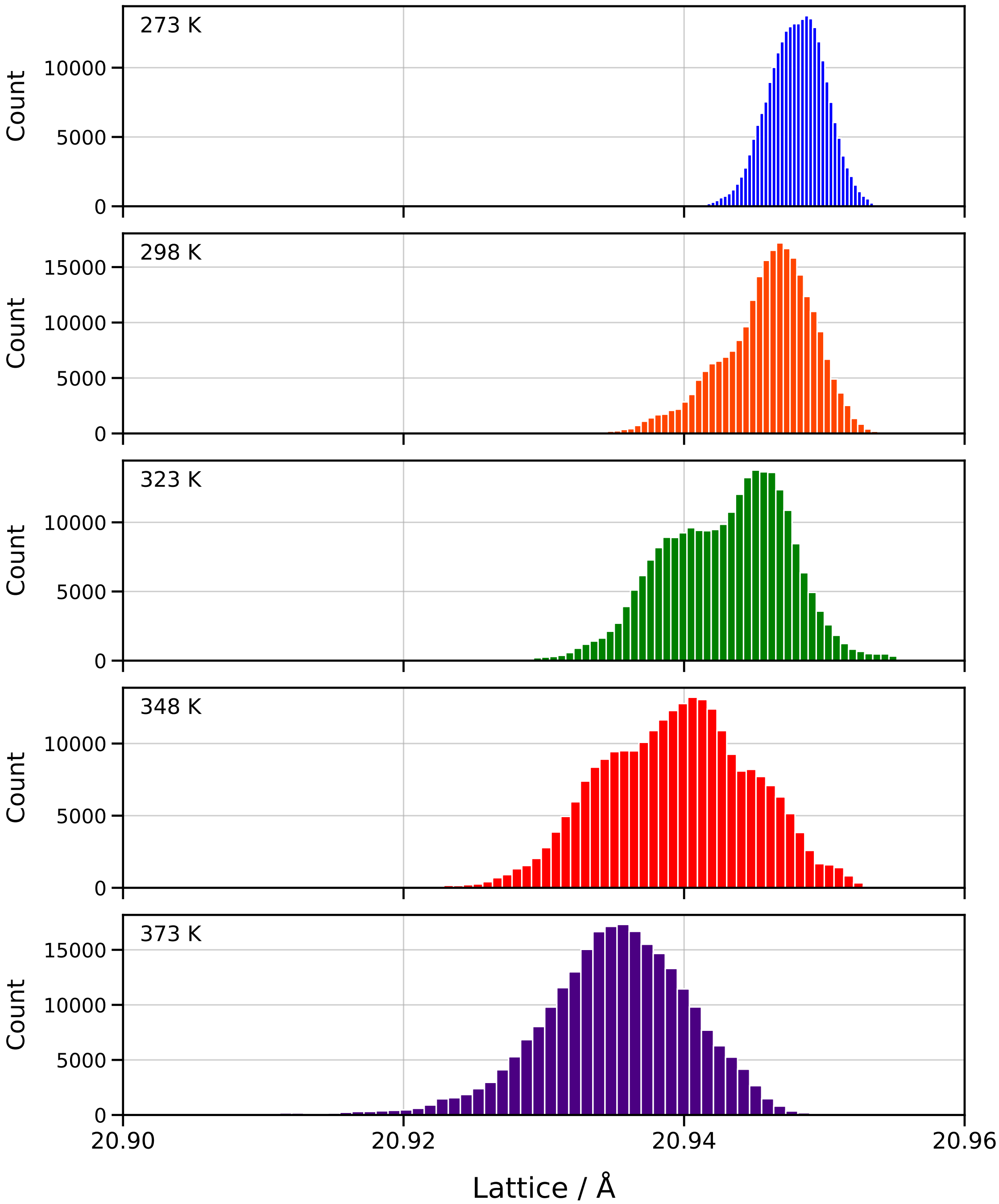

Figure S8: Distribution of the lattice parameter of UiO-66 at five equidistant temperature points in the range from 273 to 373 K, corresponding to the trajectories shown in Figure S7. The histograms have been constructed from the DFTB3/3OB MD trajectories using 50 bins each.

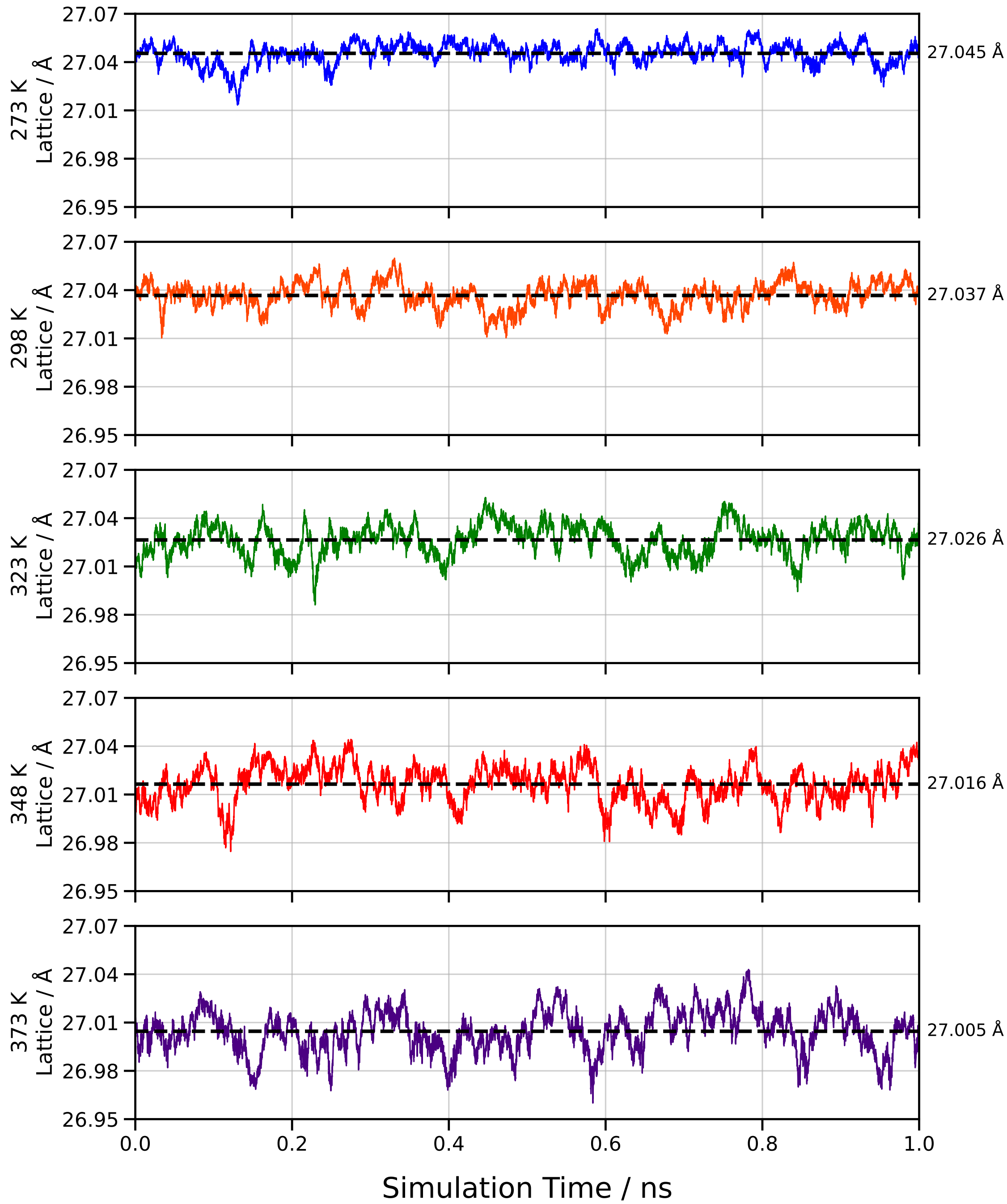

Figure S9: Time evolution of the lattice parameter of UiO-67 at five equidistant temperature points in the range from 273 to 373 K. The MD simulations have been carried out at DFTB3/3OB level of theory. Associated averages are indicated by a dashed black line.

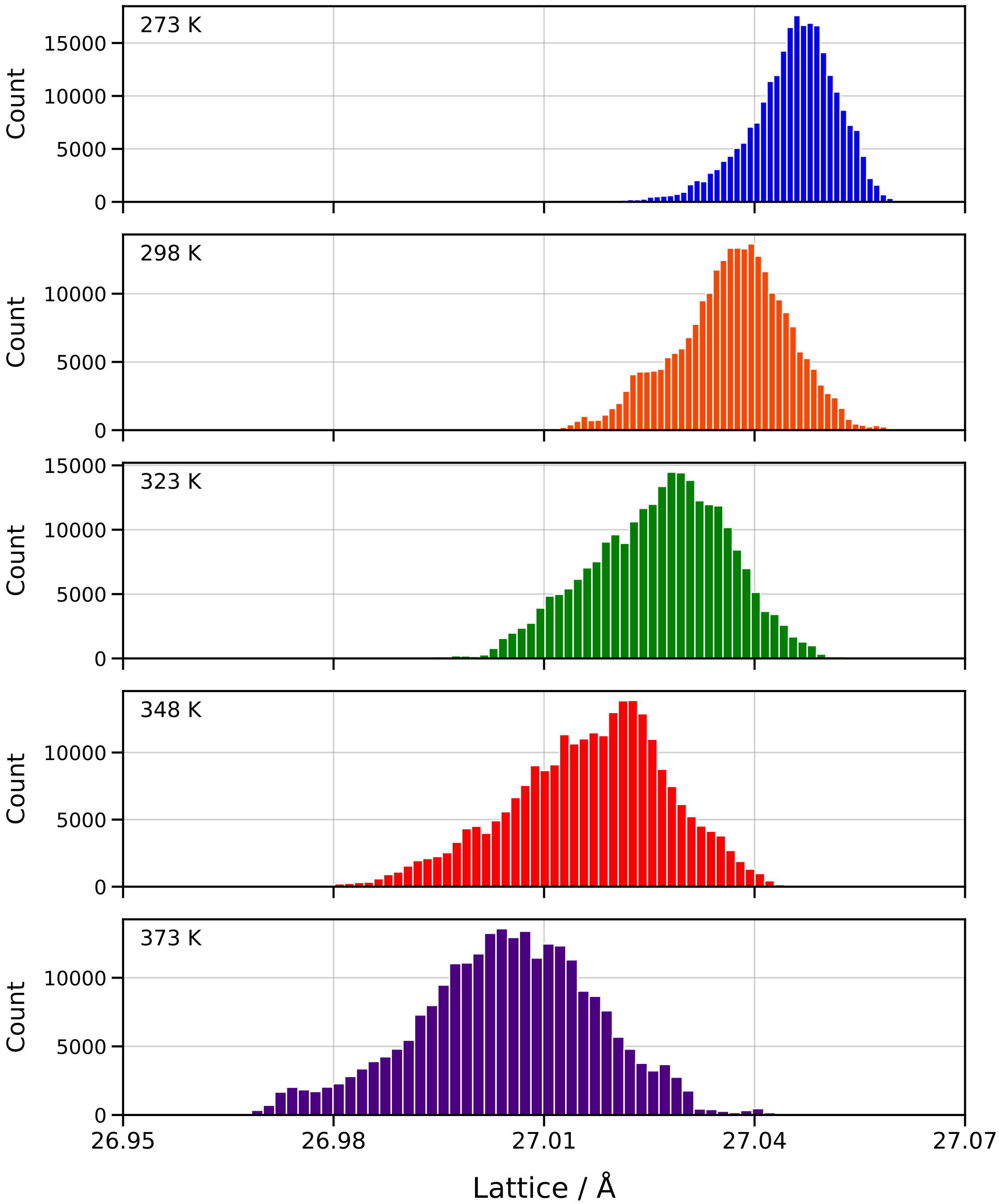

Figure S10: Distribution of the lattice parameter of UiO-67 at five equidistant temperature points in the range from 273 to 373 K, corresponding to the trajectories shown in Figure S9. The histograms have been constructed from the DFTB3/3OB MD trajectories using 50 bins each.

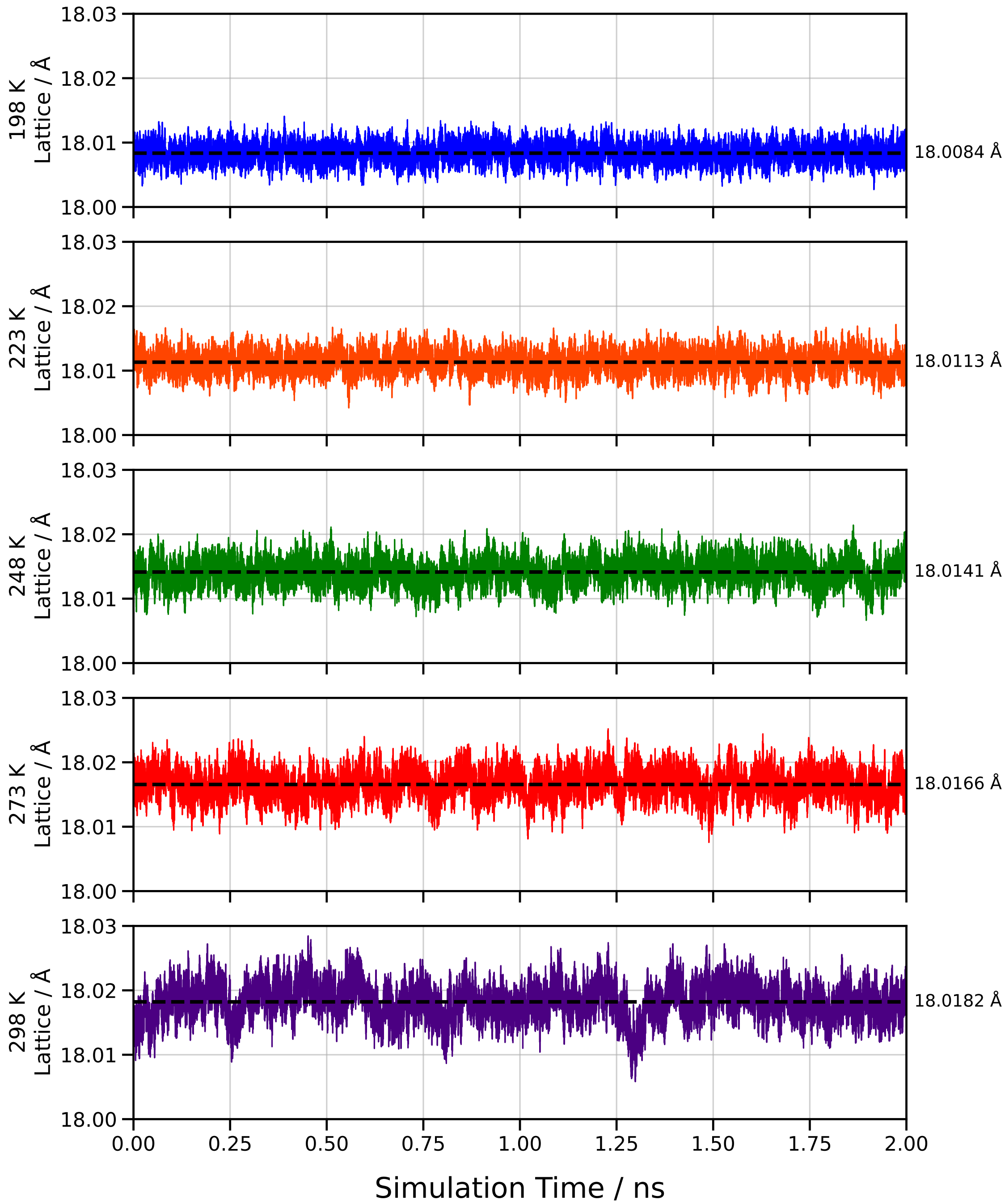

Figure S11: Time evolution of the lattice parameter of MOF-801 at five equidistant temperature points in the range from 198 to 298 K. The MD simulations have been carried out at DFTB3/3OB level of theory. Associated averages are indicated by a dashed black line.

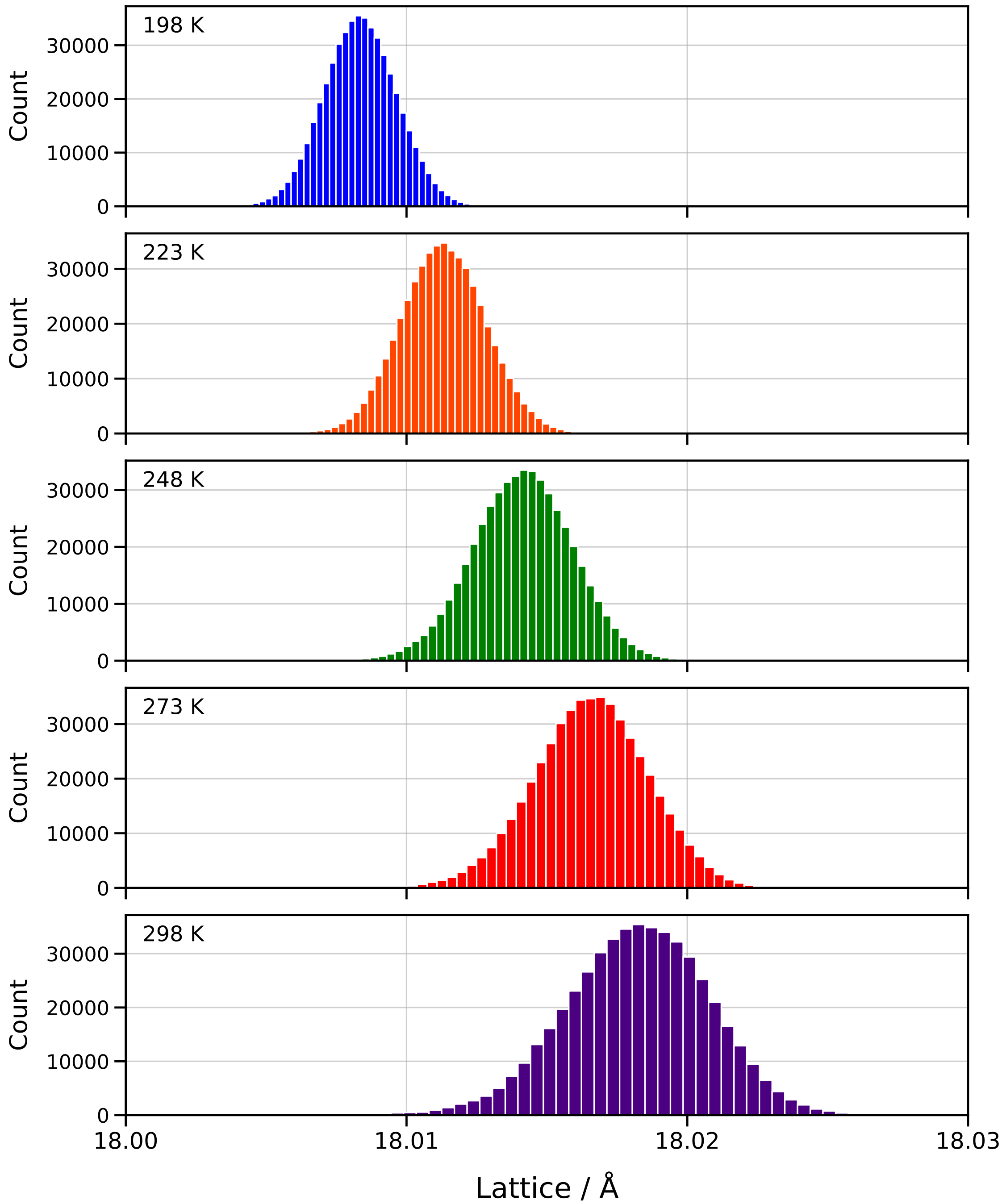

Figure S12: Distribution of the lattice parameter of MOF-801 at five equidistant temperature points in the range from 198 to 298 K, corresponding to the trajectories shown in Figure S11. The histograms have been constructed from the DFTB3/3OB MD trajectories using 50 bins each.

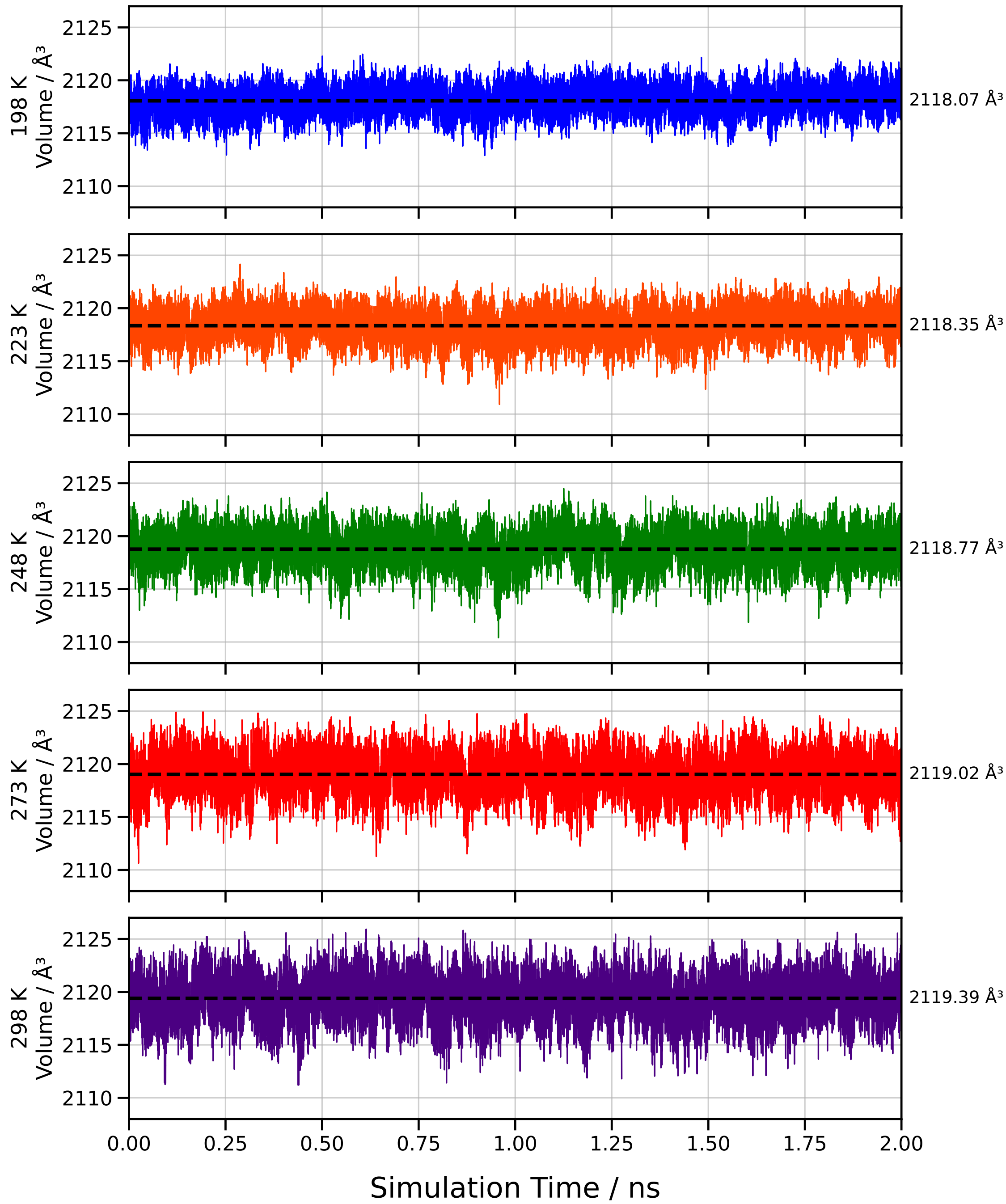

Figure S13: Time evolution of the unit cell volume of MIL-140A at five equidistant temperature points in the range from 198 to 298 K. The MD simulations have been carried out at DFTB3/3OB level of theory. Associated averages are indicated by a dashed black line.

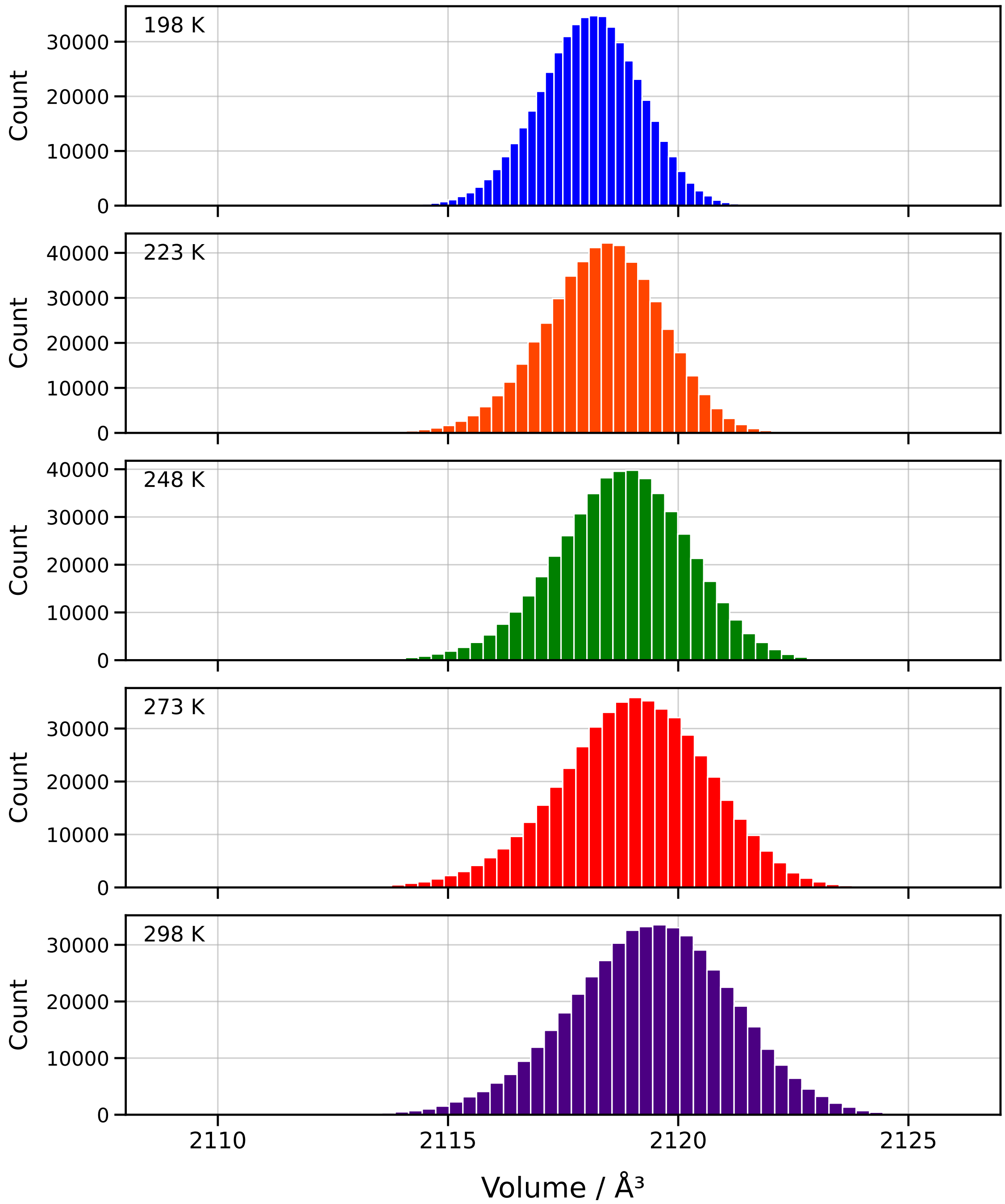

Figure S14: Distribution of the unit cell volume of MIL-140A at five equidistant temperature points in the range from 198 to 298 K, corresponding to the trajectories shown in Figure S13. The histograms have been constructed from the DFTB3/3OB MD trajectories using 50 bins each.

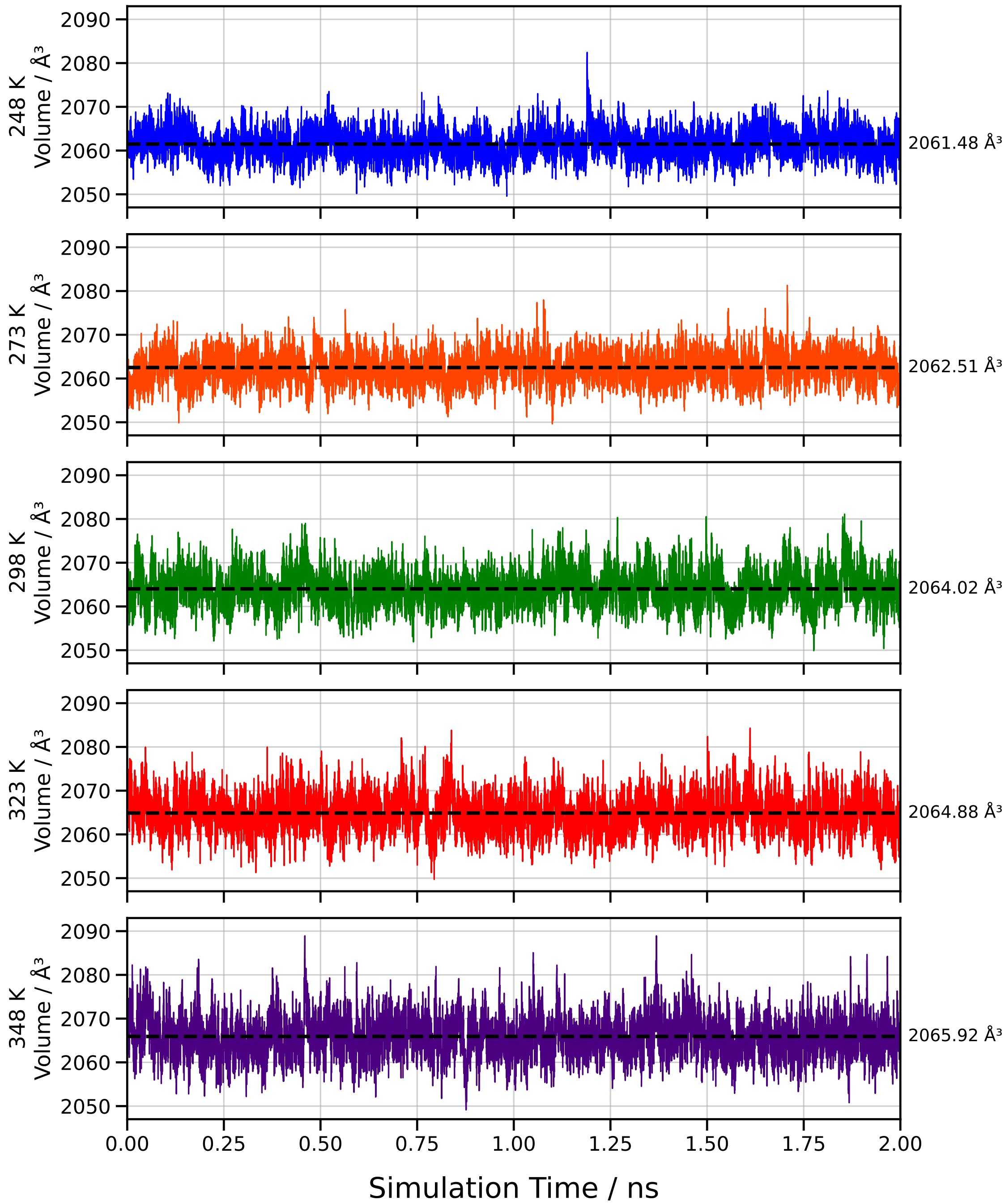

Figure S15: Time evolution of the unit cell volume of MIL-163 at five equidistant temperature points in the range from 248 to 348 K. The MD simulations have been carried out at DFTB3/3OB level of theory. Associated averages are indicated by a dashed black line.

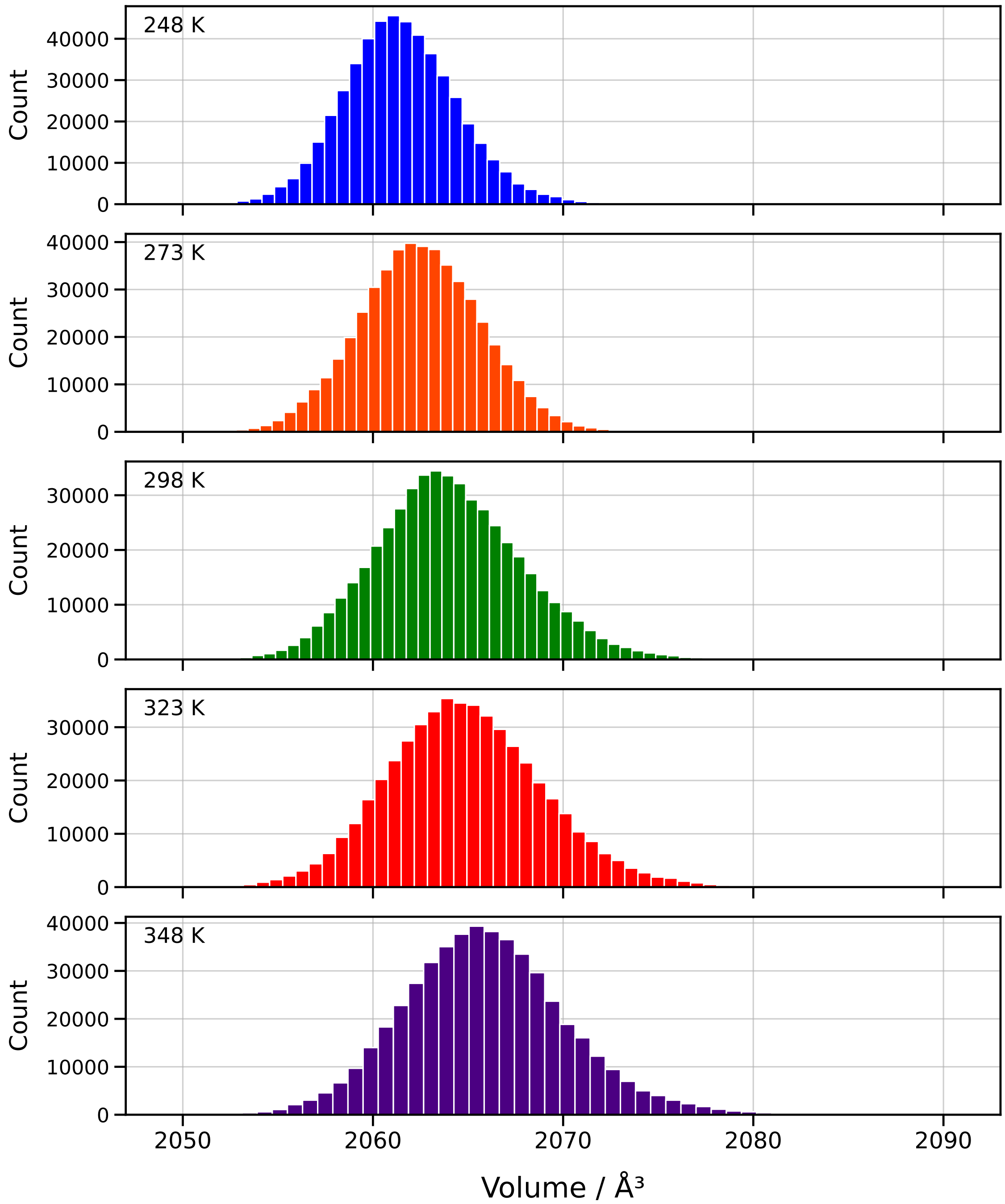

Figure S16: Distribution of the unit cell volume of MIL-163 at five equidistant temperature points in the range from 248 to 348 K, corresponding to the trajectories shown in Figure S15. The histograms have been constructed from the DFTB3/3OB MD trajectories using 50 bins each.

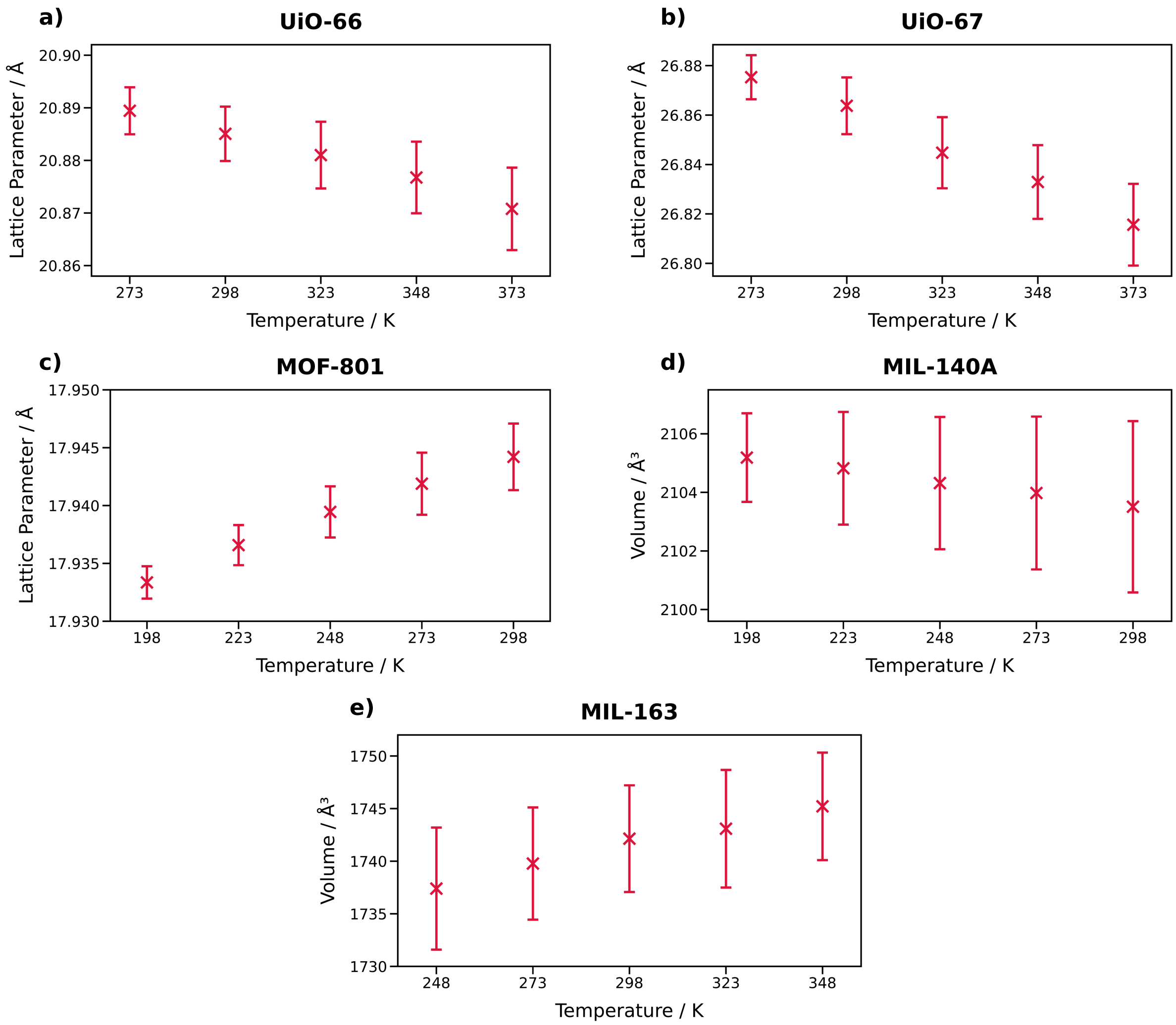

Figure S17: Lattice parameter as a function of temperature for a) UiO-66, b) UiO-67, and c) MOF-801, and unit cell volume as a function of temperature for d) MIL-140A and e) MIL-163, as determined from MACE-DAC-1 MD simulations.

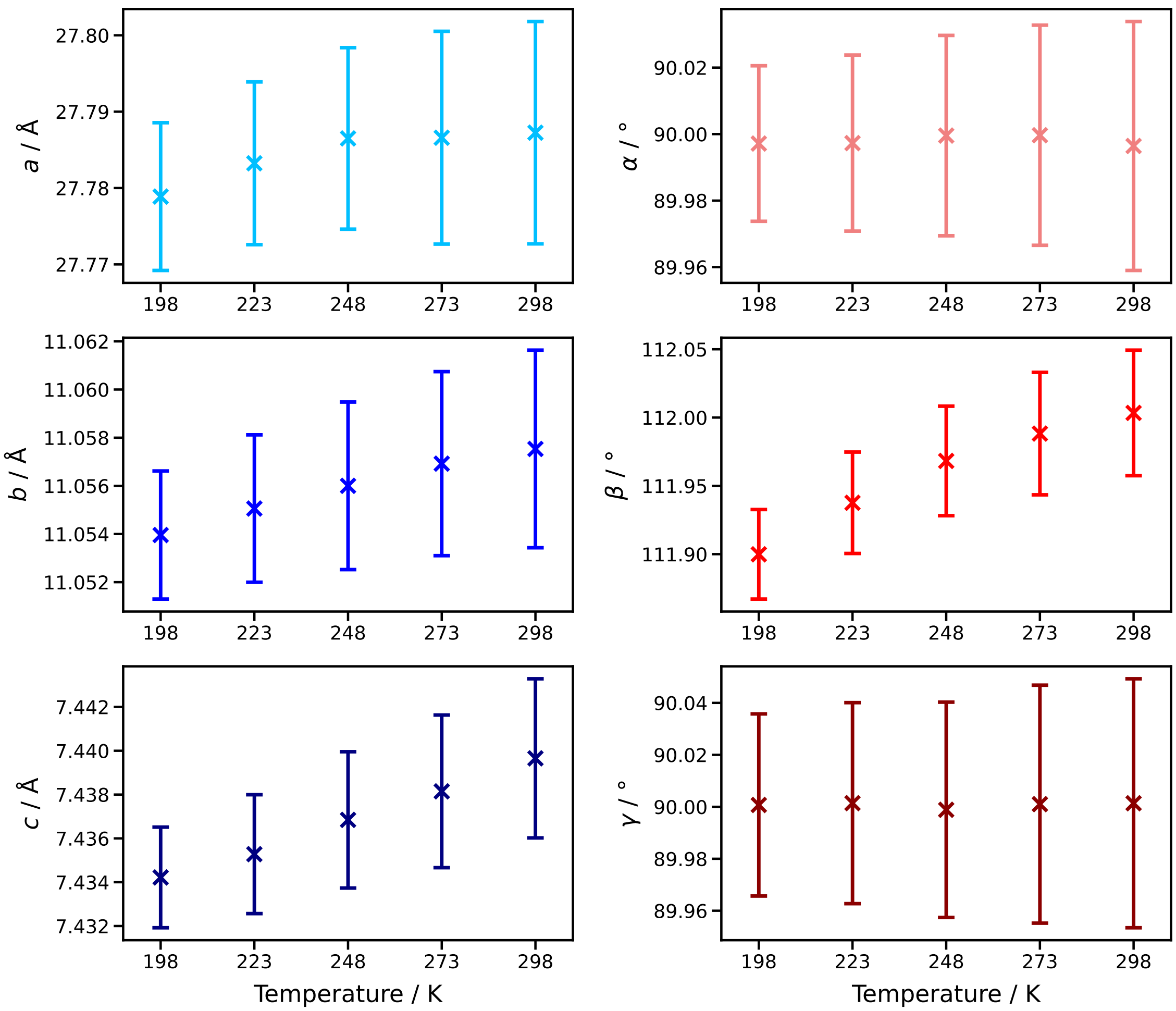

Figure S18: Temperature dependence of the lattice parameters a, b, c, α, β, and γ of MIL-140A, as determined from five independent 2 ns DFTB3/3OB MD simulations. Error bars indicate the standard deviation of the respective time-averaged quantities.

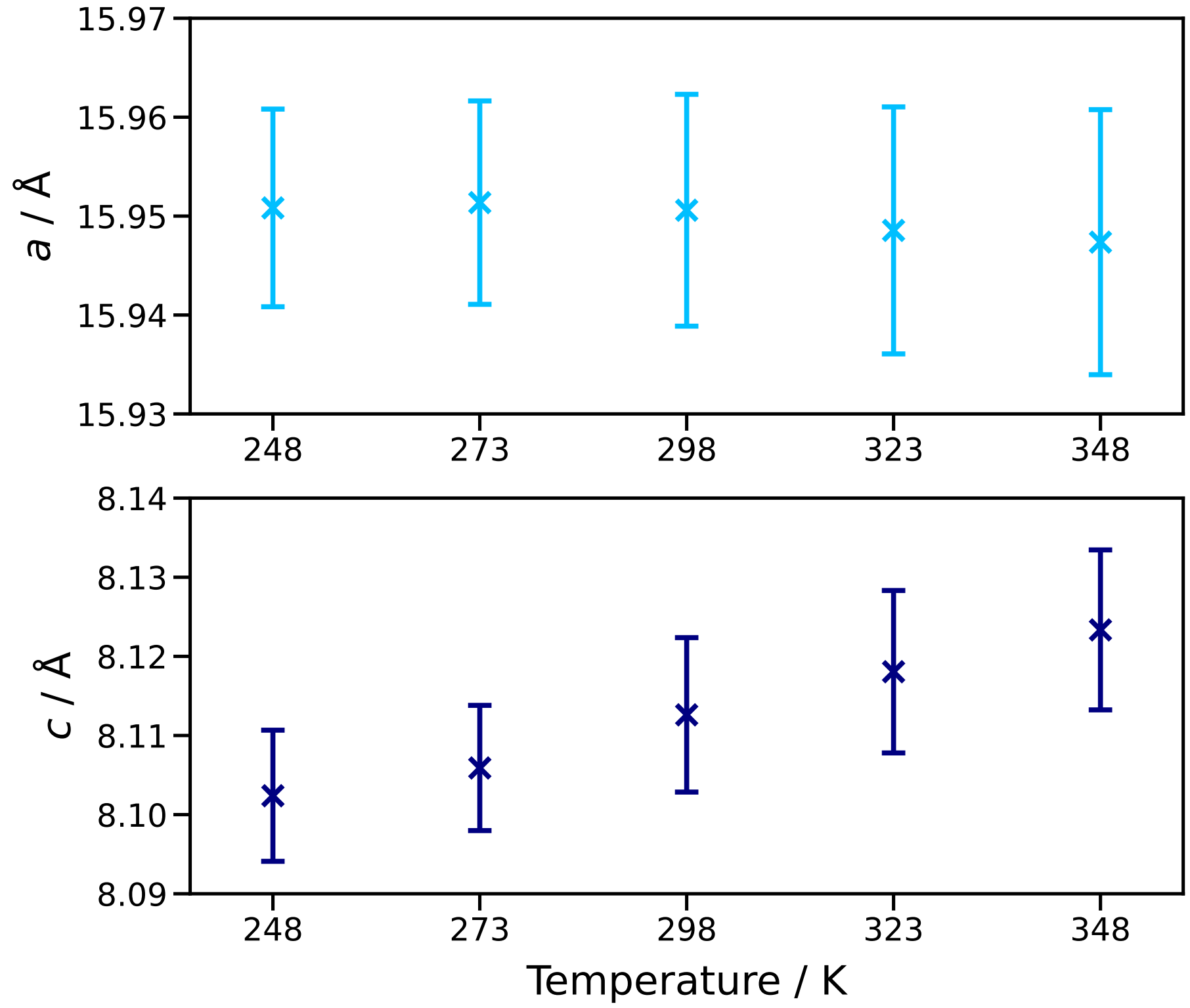

Figure S19: Temperature dependence of the lattice parameters a and c of MIL-163, as determined from five independent 2 ns DFTB3/3OB MD simulations. Error bars indicate the standard deviation of the respective time-averaged quantities.

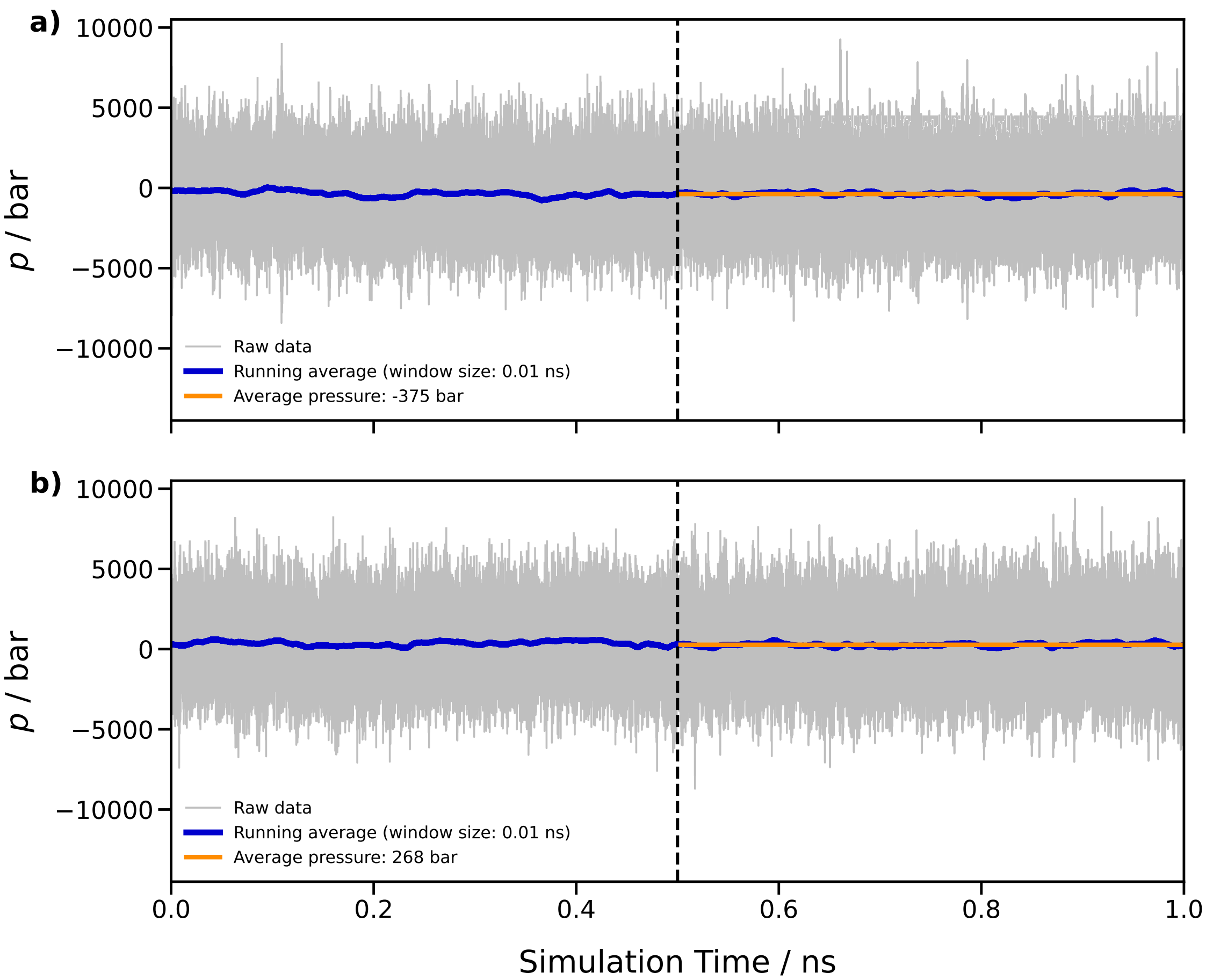

Figure S20: Time evolution of the pressure during the two *NVT* simulations of UiO-66 at DFTB3/3OB level of theory at volumes 0.1 % above a) and below b) the average volume at 298 K and 1.013 bar. The blue lines represent the associated running average over a window size of 0.01 ns. The dashed vertical lines at 0.5 ns mark the starting point for calculating the average pressures of -375 and 268 bar, indicated by the orange lines.

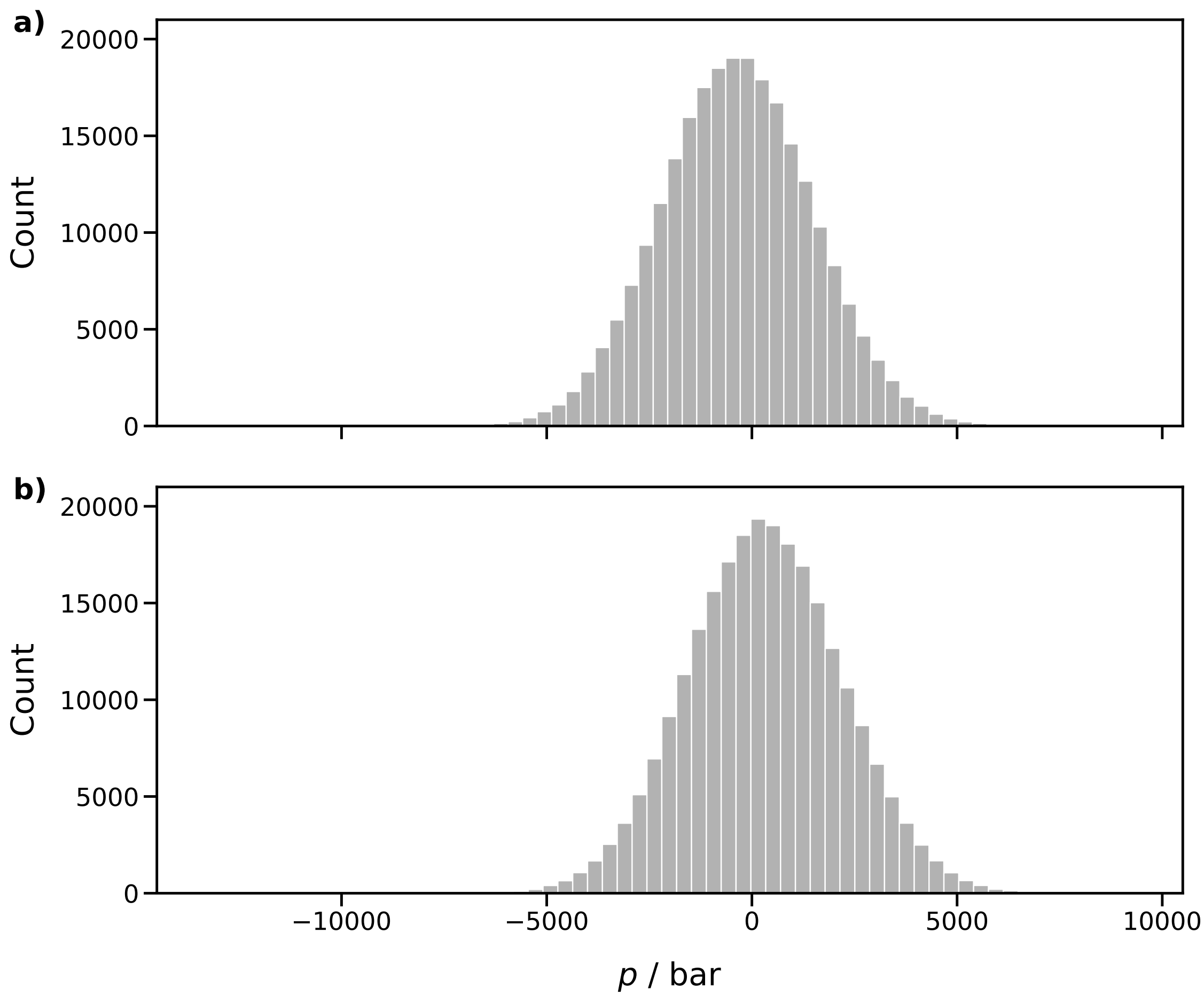

Figure S21: Distribution of the pressure during the two NVT simulations of UiO-66 at DFTB3/3OB level of theory, corresponding to the trajectories shown in Figure S20. The simulations have been performed at volumes 0.1 % above a) and below b) the average volume at 298 K and 1.013 bar. The histograms have been constructed using 50 bins each.

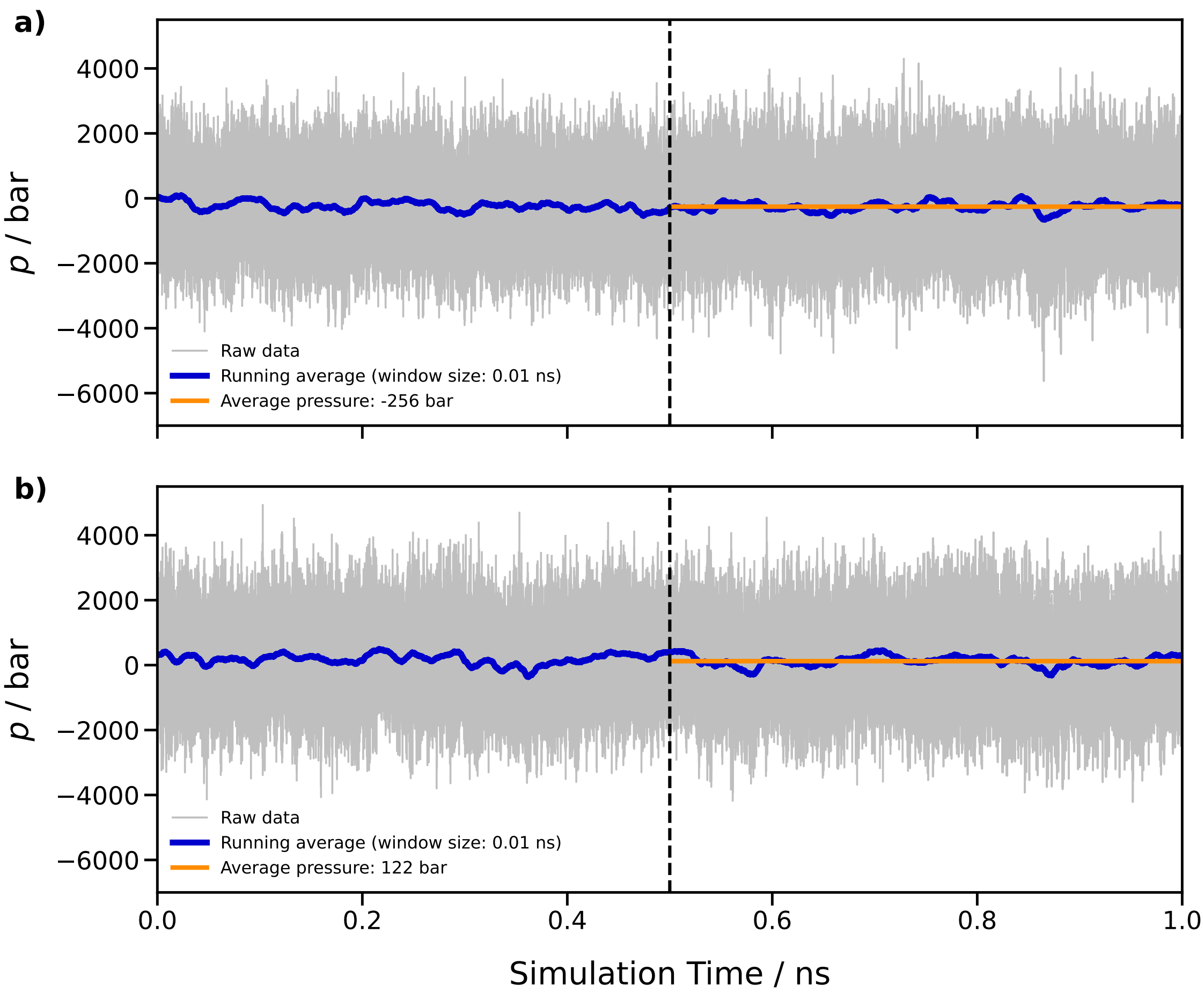

Figure S22: Time evolution of the pressure during the two NVT simulations of UiO-67 at DFTB3/3OB level of theory at volumes 0.1 % above a) and below b) the average volume at 298 K and 1.013 bar. The blue lines represent the associated running average over a window size of 0.01 ns. The dashed vertical lines at 0.5 ns mark the starting point for calculating the average pressures of -256 and 122 bar, indicated by the orange lines.

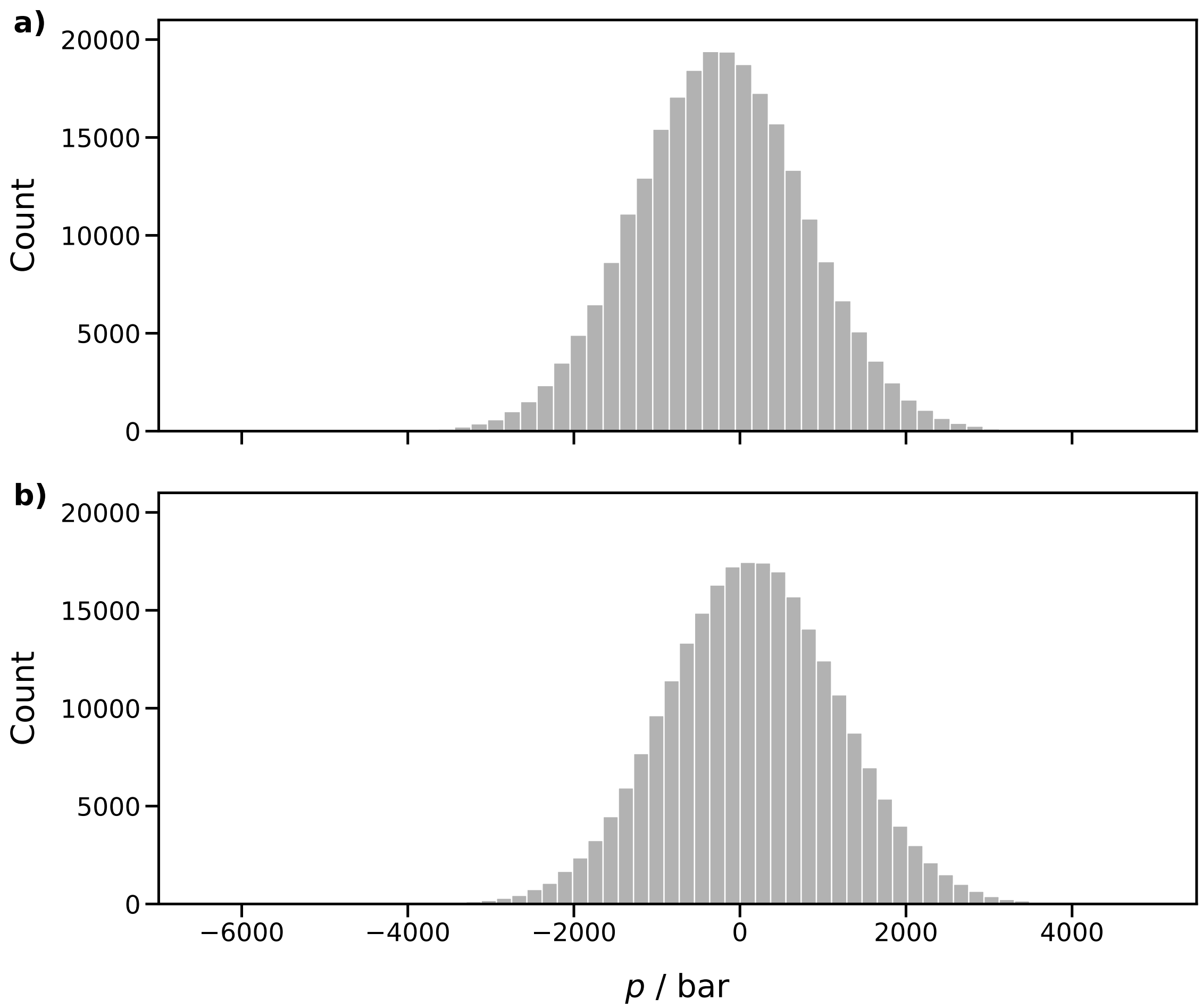

Figure S23: Distribution of the pressure during the two NVT simulations of UiO-67 at DFTB3/3OB level of theory, corresponding to the trajectories shown in Figure S22. The simulations have been performed at volumes 0.1 % above a) and below b) the average volume at 298 K and 1.013 bar. The histograms have been constructed using 50 bins each.

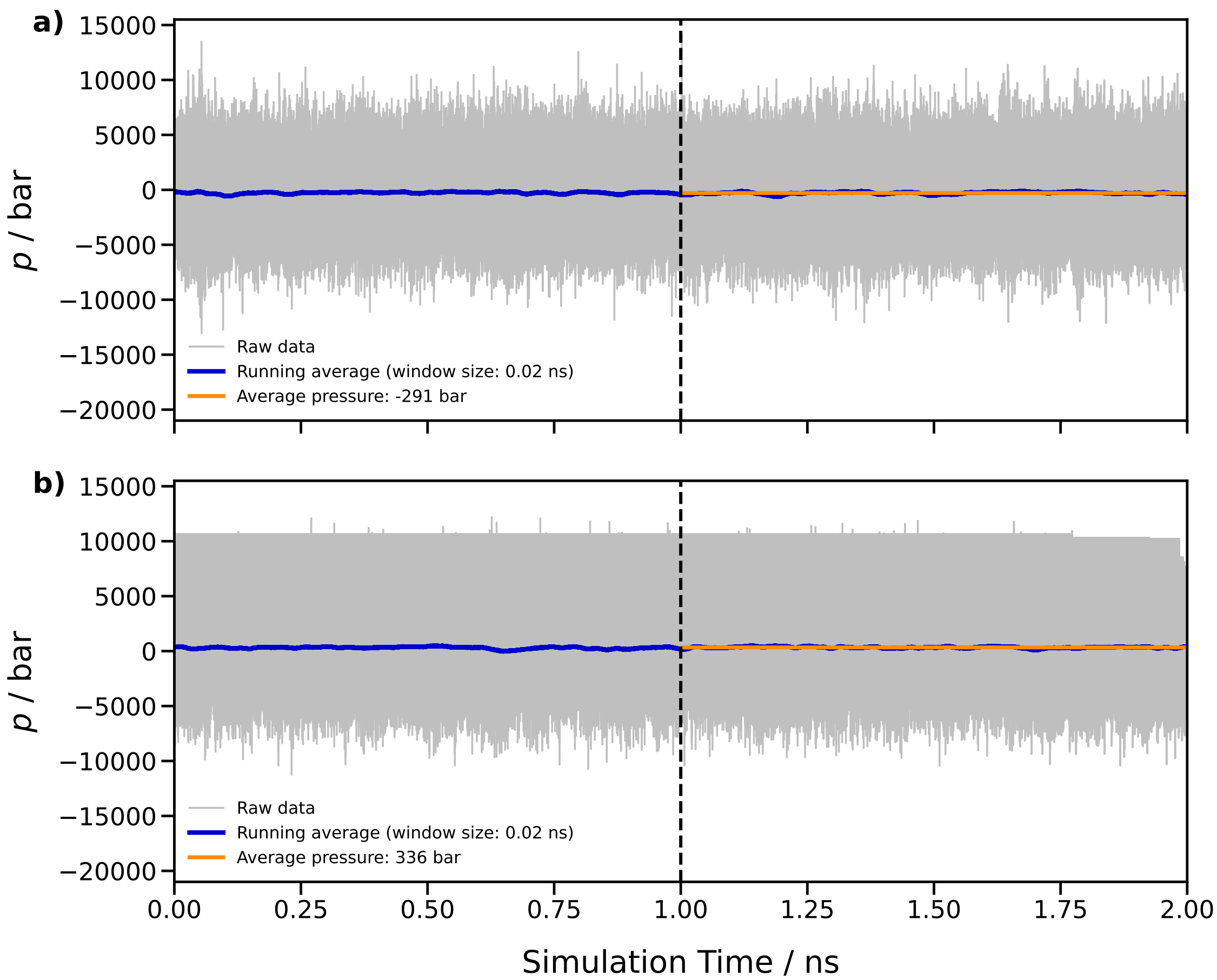

Figure S24: Time evolution of the pressure during the two *NVT* simulations of MOF-801 at DFTB3/3OB level of theory at volumes 0.1 % above a) and below b) the average volume at 298 K and 1.013 bar. The blue lines represent the associated running average over a window size of 0.02 ns. The dashed vertical lines at 1.0 ns mark the starting point for calculating the average pressures of -291 and 336 bar, indicated by the orange lines.

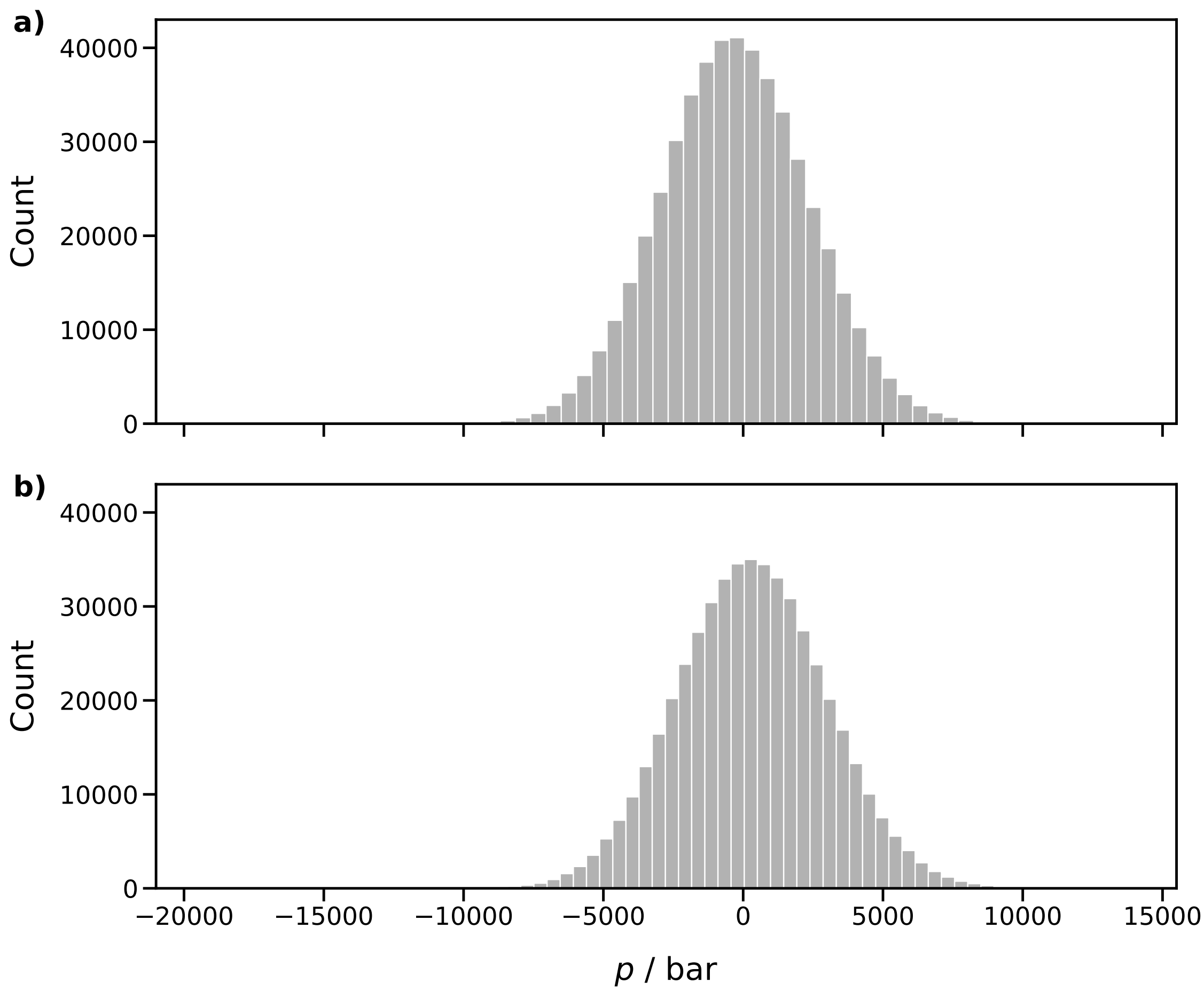

Figure S25: Distribution of the pressure during the two *NVT* simulations of MOF-801 at DFTB3/3OB level of theory, corresponding to the trajectories shown in Figure S24. The simulations have been performed at volumes 0.1 % above a) and below b) the average volume at 298 K and 1.013 bar. The histograms have been constructed using 50 bins each.

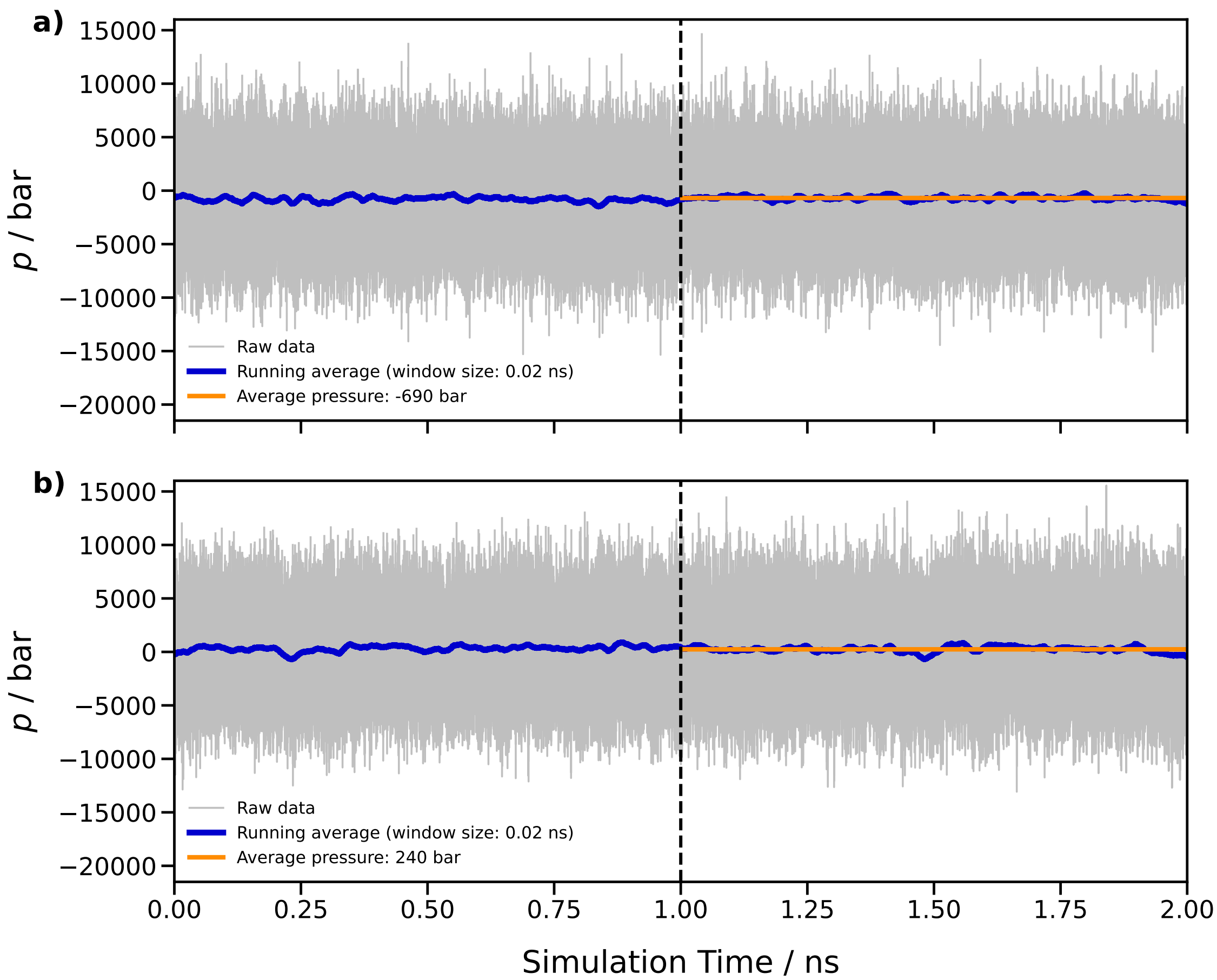

Figure S26: Time evolution of the pressure during the two NVT simulations of MIL-140A at DFTB3/3OB level of theory at volumes 0.1 % above a) and below b) the average volume at 298 K and 1.013 bar. The blue lines represent the associated running average over a window size of 0.02 ns. The dashed vertical lines at 1.0 ns mark the starting point for calculating the average pressures of -690 and 240 bar, indicated by the orange lines.

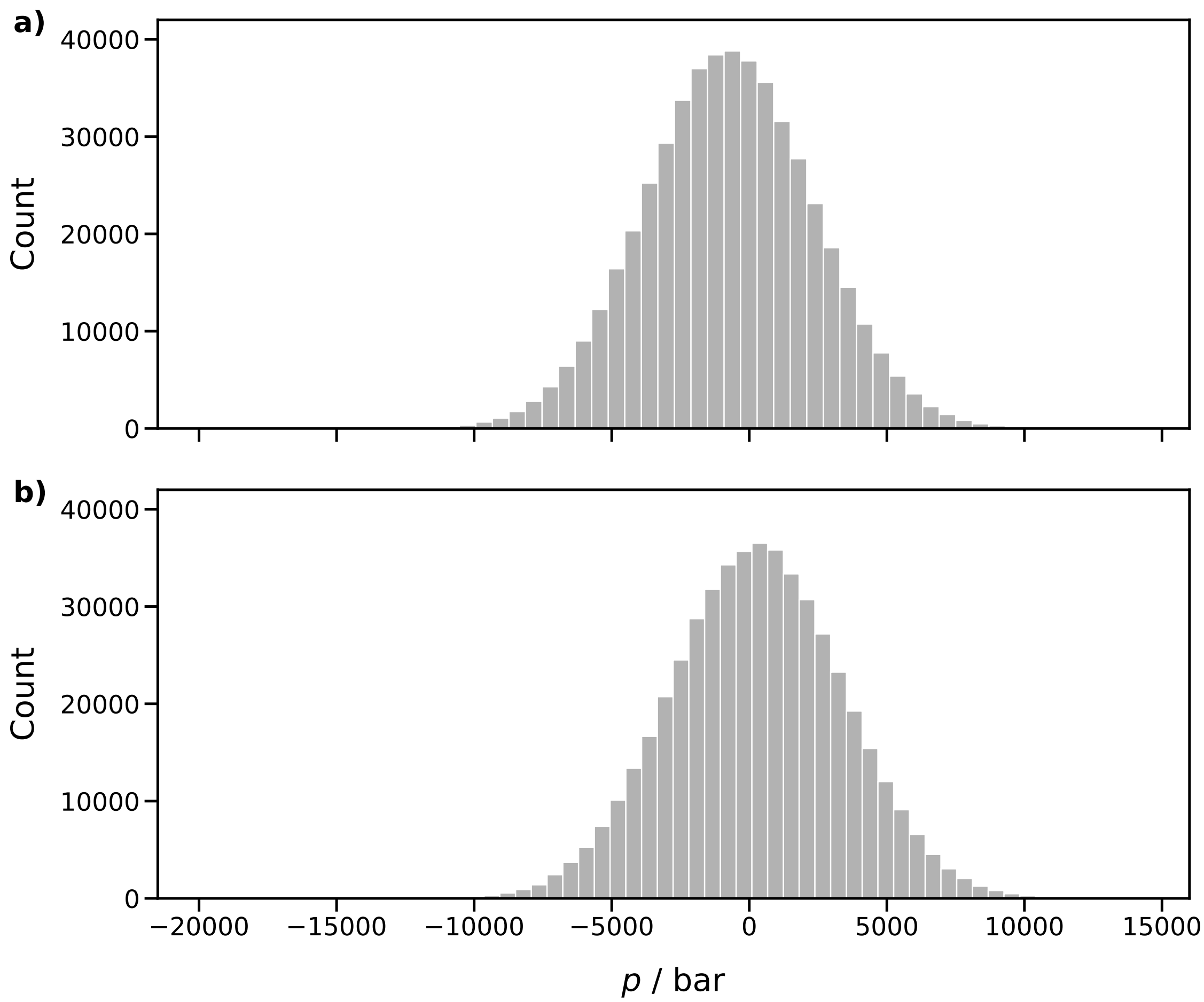

Figure S27: Distribution of the pressure during the two NVT simulations of MIL-140A at DFTB3/3OB level of theory, corresponding to the trajectories shown in Figure S26. The simulations have been performed at volumes 0.1 % above a) and below b) the average volume at 298 K and 1.013 bar. The histograms have been constructed using 50 bins each.

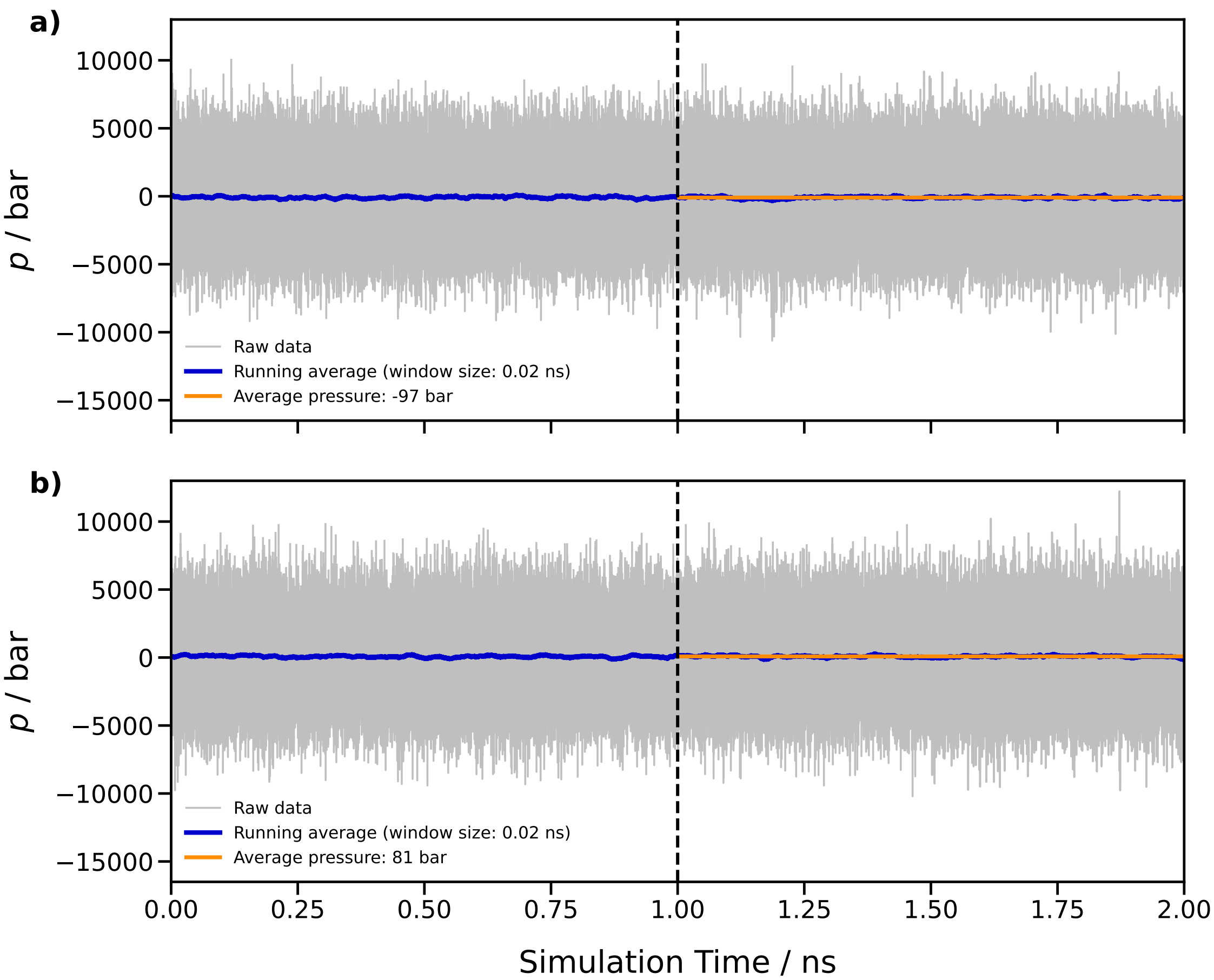

Figure S28: Time evolution of the pressure during the two *NVT* simulations of MIL-163 at DFTB3/3OB level of theory at volumes 0.1 % above a) and below b) the average volume at 298 K and 1.013 bar. The blue lines represent the associated running average over a window size of 0.02 ns. The dashed vertical lines at 1.0 ns mark the starting point for calculating the average pressures of -97 and 81 bar, indicated by the orange lines.

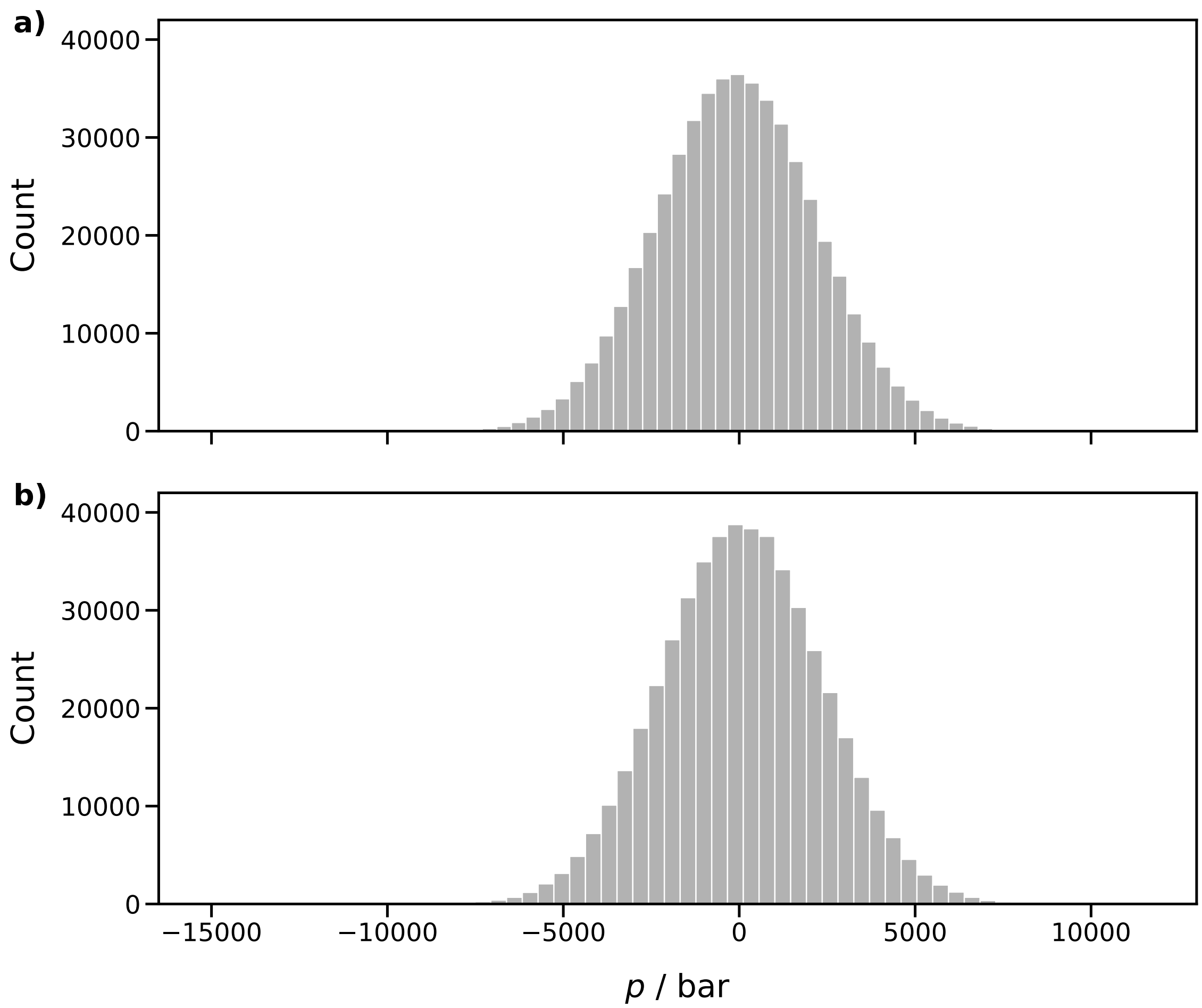

Figure S29: Distribution of the pressure during the two *NVT* simulations of MIL-163 at DFTB3/3OB level of theory, corresponding to the trajectories shown in Figure S28. The simulations have been performed at volumes 0.1 % above a) and below b) the average volume at 298 K and 1.013 bar. The histograms have been constructed using 50 bins each.
